# Identification and Characterization of the Two Glycosyltransferases Required for the Polymerization of the HS:1 Serotype Capsular Polysaccharide of *Campylobacter jejuni* G1

**DOI:** 10.1021/acs.biochem.4c00803

**Published:** 2025-02-28

**Authors:** Ronnie Bourland, Tamari Narindoshvili, Frank M. Raushel

**Affiliations:** † Department of Biochemistry & Biophysics, Texas A&M University, College Station, Texas 77843, United States; ‡ Department of Chemistry, Texas A&M University, College Station, Texas 77843, United States

## Abstract

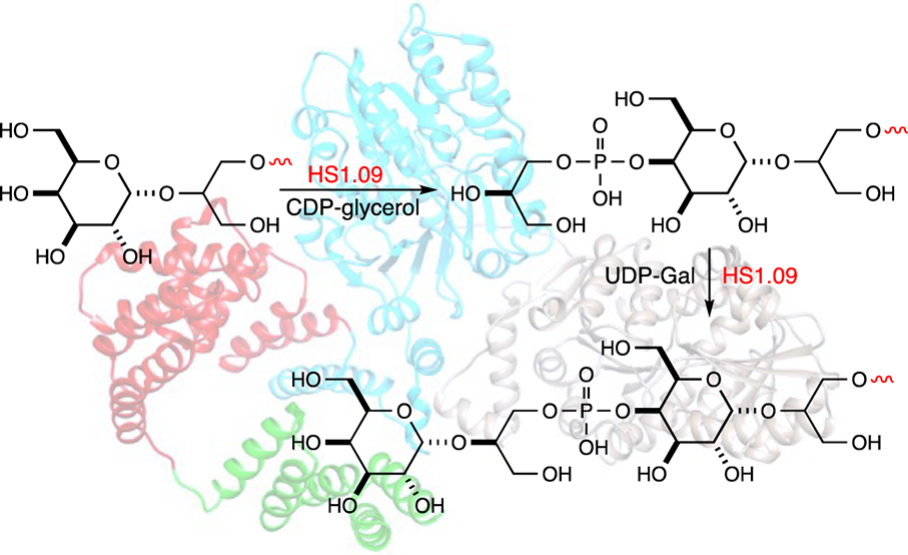

*Campylobacter jejuni* is a Gram-negative pathogenic bacterium commonly found in poultry and is the leading cause of gastrointestinal infections in the United States. Similar to other Gram-negative bacteria, *C. jejuni* possesses an extracellular carbohydrate-based capsular polysaccharide (CPS) composed of repeating units of monosaccharides bound via glycosidic linkages. The gene cluster for serotype 1 (HS:1) of *C. jejuni* contains 13 different genes required for the production and presentation of the CPS. Each repeating unit within the HS:1 CPS structure contains a backbone of glycerol phosphate and d-galactose. Here, the enzyme HS1.11 was shown to catalyze the formation of CDP-(2*R*)-glycerol from MgCTP and l-glycerol-3-phosphate. HS1.09 was found to be a multidomain protein that catalyzes the polymerization of l-glycerol-3-phosphate and d-galactose using UDP-d-galactose and CDP-(2*R*)-glycerol as substrates. The domain of HS1.09 that extends from residues 286 to 703 was shown to catalyze the transfer of l-glycerol-P from CDP-glycerol to the hydroxyl group at C4 of the d-galactose moiety at the nonreducing end of the growing oligosaccharide. The transfer of d-galactose to the C2 hydroxyl group of the glycerol-phosphate moiety was shown to be catalyzed with retention of configuration by the domain of HS1.09 that extends from residues 704 to 1095. Primers as short as a single d-galactoside were accepted as initial substrates. Oligosaccharide products were isolated by ion exchange chromatography and identified by high-resolution ESI-mass spectrometry and NMR spectroscopy.

## Introduction

*Campylobacter jejuni* is a Gram-negative pathogenic bacterium commonly found in poultry and is the leading cause of gastrointestinal infections in the United States and Europe.^1−3^*C. jejuni* is commensal in chickens and infections are spread to humans through the consumption of undercooked and contaminated poultry products.^1−3^ The pathogenicity of *C. jejuni* is attributed to its adaptability, host immune system evasion, and increasing antibiotic resistance.^4^ Common symptoms of campylobacteriosis include intestinal inflammation, fever, diarrhea, and vomiting.^5^ The detrimental effects from *C. jejuni* infections are thought to arise from a bacterial secreted genotoxin called cytolethal distending toxin (CDT).^6^ CDT is derived from the bacterial expression and secretion of the heterotrimeric protein complex of CdtA, CdtB, and CdtC.^6^ In rare cases, *C. jejuni* infections can lead to the development of autoimmune diseases including Guillain-Barré Syndrome and Miller Fisher Syndrome.^5,7^ The clinical symptoms for both diseases are muscle weakness and progressive paralysis due to nerve demyelination.^7,8^

Unfortunately, there are no FDA-approved vaccines for any strain or serotype of *C. jejuni*.^9^ However, a successful and widely used glycoconjugate vaccine has been developed against Gram-negative *Haemophilus influenzae* serotype b (Hib) that targets the exterior capsular polysaccharide (CPS).^10^ The implementation of the Hib glycoconjugate vaccine required large-scale pathogen cultivation and direct CPS isolation from pathogen cultures, leading to high production costs and limited availability.^11−13^ Recent studies of *H. influenzae*, *Neisseria meningitidis*, and *Actinobacillus pleuropneumoniae* have focused on a new method for antigen synthesis using recombinant enzymes to produce the CPS *in vitro* for subsequent attenuation to a carrier protein.^12,14^ The Hib vaccine may function as a model for the development of a comparable glycoconjugate vaccine for *C. jejuni*.

**Figure 1:**
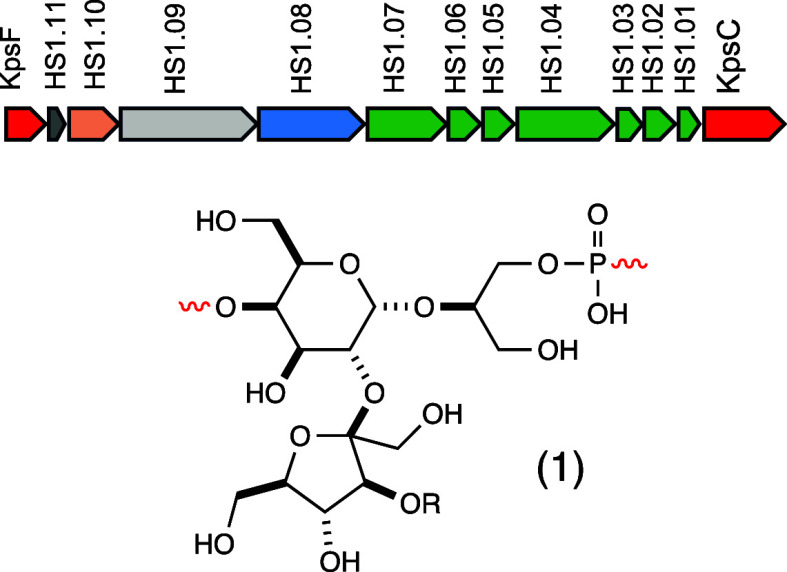
Gene cluster for the biosynthesis of the capsular polysaccharide in the HS:1 serotype of *C. jejuni* G1 (top). Structure of the repeating unit within the capsular polysaccharide of the HS:1 serotype of *C. jejuni* (bottom) where R denotes a methyl phosphoramidate modification. The genes colored green encode enzymes for the biosynthesis of the methyl phosphoramidate modification. The blue-colored gene is responsible for the fructose modification to the CPS. The pink-colored gene is presumably responsible for the initiation of polysaccharide formation to the Kdo-linker. The light gray gene is likely responsible for the polymerization of glycerol-phosphate and d-galactose, while the dark gray colored gene is responsible for the synthesis of CDP-glycerol.

Like other Gram-negative bacteria, *C. jejuni* possesses a CPS structure composed of a repeating polysaccharide that is anchored to the membrane surface through a poly-Kdo (3-deoxy-d-manno-oct-2-ulosonic acid) linker.^15^ The composition of *C. jejuni* CPS structures are highly variable between different strains and serotypes with 12 known CPS structures from more than 33 different serotypes.^16−18^ The gene cluster for CPS formation in the HS:1 serotype is shown in Figure 1.^19,20^ Among the genetically characterized gene clusters for CPS formation in *C. jejuni*, this is perhaps the least complicated in that it encodes for only 11 enzymes between the genes for KpsC and KpsF. The repeating structural motif unit within the CPS from this serotype contains a backbone of (2*R*)-glycerol-phosphate and d-galactose. The galactose moiety is further linked at C2 with d-fructose that is further decorated with a methyl phosphoramidate group (Figure 1).^19,20^

The focus of this investigation is the identification and characterization of those enzymes required for the polymerization of the repeating disaccharide containing (2*R*)-glycerol-P and d-galactose within the CPS of the HS:1 serotype. The genes for these two activities are expected to be found within the gene cluster for CPS biosynthesis shown in Figure 1. The enzymes denoted as HS1.01 through HS1.07 (highlighted in green) are required for the synthesis and transfer of the methyl phosphoramidate group to the d-fructose moiety in the CPS, based on genetic and enzymatic characterization of homologous enzymes identified from the HS:2 serotype.^21−24^ A recent genetic study of the HS:1 serotype suggested that HS1.08 (highlighted in blue) is required for the activation and transfer of d-fructose to the growing polysaccharide chain.^19,20^ The catalytic function of HS1.10 (highlighted in pink) is currently unknown, but it is likely involved in the initiation of glycosyl transfer to the poly-Kdo linker.^25^ A recent study analyzing the initiation of CPS polymerization in the Gram-negative bacterium, *A. pleuropneumoniae* serotype 3, demonstrated the requirement for two different transition transferases, Cps3C and Cps3A, to attach multiple glycerol-phosphate moieties to the poly-Kdo linker.^25^ HS1.10 is homologous to Cps3C and thus this enzyme is predicted to be involved in the initiation of polysaccharide formation in the HS:1 serotype of *C. jejuni*. The smallest protein encoded by this gene cluster, HS1.11 (colored dark gray), is 45% identical to that of l-glycerol-3-P cytidylyltransferase from *Bacillus subtilis* and thus this enzyme is expected to be required for the activation of l-glycerol-3-P to generate CDP-(2*R*)-glycerol needed for the polymerization of the CPS.^26^ The remaining enzyme in the gene cluster (HS1.09) is a large multidomain protein of 1095 amino acids (colored light gray). One of the domains has a TagF-like structural fold and another has a GT-B glycosyltransferase fold and thus this multifunctional enzyme is therefore postulated to catalyze the transfer of glycerol-P and d-galactose to the growing capsular polysaccharide.^27,28^ In this paper we demonstrate that HS1.11 catalyzes the synthesis of CDP-(2*R*)-glycerol and that HS1.09 catalyzes the polymerization of glycerol-phosphate and d-galactose using CPD-(2*R*)-glycerol and UDP-galactose as substrates.

## Materials and Methods

### Materials

**Figure 2:**
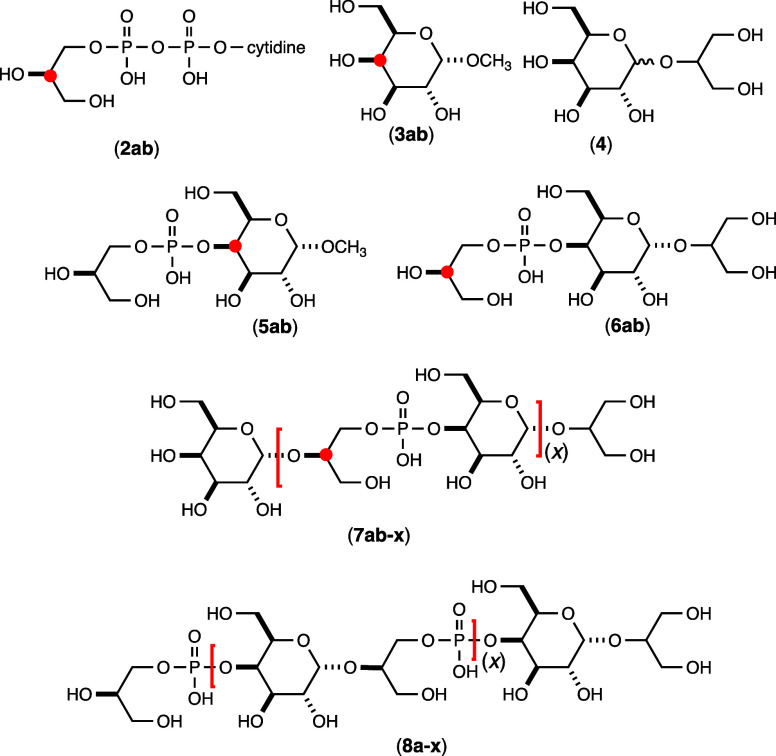
Chemical structures of the substrates and products used in this investigation. Those compounds highlighted with a closed red circle indicate the position of a ^13^C-label. The unlabeled compound is designated with an “**a**” while the labeled compound is designated with a “**b**”. For those compounds highlighted with red brackets the (**x**) indicates the number of repeating units within the bracket.

All chemicals, buffers, and coupling enzymes were purchased from MilliporeSigma, unless otherwise specified. The ^13^C-labeled compounds were purchased from Omicron Biochemicals. Shrimp alkaline phosphatase was obtained from New England Biolabs. All prepacked chromatography columns, protein concentrators, and filters were purchased from Cytiva. The structures of compounds prepared for this investigation are provided in Figure 2. The preparative methods for the synthesis of compounds **3b** and **4** are provided in the Supporting Information.

### Purification of l-Glycerol-3-phosphate Cytidylyltransferase (GCT)

The gene for HS1.11 (UniProt: Q5M6N5) in the biosynthetic gene cluster of *C. jejuni* from serotype HS:1 was chemically synthesized in a pET-29b(+) vector by Twist Biosciences with a C-terminal polyhistidine purification tag. The plasmid was transformed through electroporation into *E. coli* BL21(DE3) electrocompetent cells and a single colony was selected from cells grown at 37 °C on LB agar plates supplemented with 50 μg/mL kanamycin. The cells were used to inoculate 5 mL cultures of LB medium supplemented with 50 μg/mL kanamycin and grown overnight at 37 °C. The 5 mL cultures were subsequently used to inoculate 1.0 L of fresh LB medium supplemented with 50 μg/mL kanamycin and grown at 37 °C until the OD_600_ was ∼0.7. The cultures were induced with 1.0 mM isopropyl β-thiogalactoside (IPTG) at 37 °C for 4 h before harvesting the cells by centrifugation. The cell pellet was resuspended in lysis buffer (50 mM Bis-tris propane, pH 9.0, 500 mM NaCl, 1.0 mM DTT, 20 mM imidazole, 10% glycerol, 0.01% TritonX-100) and 1.0 mg of DNase before sonication. The insoluble fraction of the lysate was pelleted via centrifugation at 4 °C. The supernatant fluid was passed through a 0.45 μm filter before loading onto a prepacked 5 mL HisTrap HP nickel affinity column. The protein was eluted with a 30-column gradient of elution buffer (50 mM Bis-tris propane, pH 9.0, 500 mM NaCl, 1.0 mM DTT, 500 mM imidazole, 10% glycerol, 0.01% TritonX-100). The fractions containing the protein were pooled, and excess imidazole was removed with dialysis buffer (50 mM Bis-tris propane, pH 9.0, 500 mM NaCl, 1.0 mM DTT, 10% glycerol, 0.01% TritonX-100) using a 20 mL (10 kDa MWCO) concentrator. The protein samples were flash frozen in liquid nitrogen and stored at −80 °C. Approximately 20 mg of purified protein was obtained per liter of cell culture. The amino acid sequence of the purified GCT is presented in Figure S1a.

### Preparation of CDP-α-(2*R*)-Glycerol (**2a**)

CDP-glycerol was prepared enzymatically in a reaction of 3.0 mL containing 15 mM CTP, 15 mM l-glycerol-3-P, 17 mM MgCl_2_ in 50 mM NH_4_HCO_3_, pH 8.0. The reaction was initiated by the addition of 10 μM GCT and supplemented with 0.6 units of inorganic pyrophosphatase and allowed to incubate for 2 h at 25 °C. After the reaction was complete, 60 units of alkaline phosphatase were added to hydrolyze the remaining CTP. The enzymes were removed via passage through a 10 kDa MWCO filter and the product purified using a 5 mL HiTrap Q HP anion exchange column with a gradient of 0–500 mM NH_4_HCO_3_, pH 8.0. The concentration of the CDP-glycerol was determined using an extinction coefficient of 9,000 M^–1^ cm^–1^ at 271 nm.^29^

### Preparation of CDP-α-[2*R*-^13^C]-Glycerol (**2b**)

The isotopically labeled CDP-glycerol was synthesized enzymatically from [2*R*-^13^C]-glycerol-3-P and CTP using the conditions described above for the preparation of the unlabeled compound. The [2*R*-^13^C]-glycerol-3-P was prepared enzymatically using glycerol kinase (2 units/mL), 15 mM ATP and 75 mM [2-^13^C]-glycerol in a 2.5 mL reaction mixture containing 50 mM NH_4_HCO_3_, pH 8.0 and 17 mM MgCl_2_ at 25 °C for 4 h. At the end of the reaction the glycerol kinase was removed by filtration.

### Characterization of l-Glycerol-3-phosphate Cytidylyltransferase

The catalytic properties of GCT were tested with different nucleotide triphosphates and acceptor substrates. Product formation was determined as a function of time by measuring the concentration of XDP-glycerol using a HiTrap Q HP anion exchange column in 20 mM HEPES, pH 8.0, with a gradient of KCl and monitoring the absorbance at 255 nm. The reactions were conducted in 50 mM HEPES, pH 8.0, with 4.0 mM MgCl_2_ at 25 °C. The nucleotide triphosphates (ATP, GTP, CTP, and UTP) were tested at 2.0 mM with 5.0 mM l-glycerol-3-P using 15–150 nM GCT in the presence of 0.2 units/mL of pyrophosphatase. d-Glycerol-3-P was tested at 5.0 mM in the presence of 2.0 mM CTP with 520 nM GCT.

### Purification of the Glycerol-P Transferase Domain of HS1.09

The gene for HS1.09 (UniProt: Q5M6N7) was truncated to include only the putative glycerol-P transferase domain that extends from amino acids 286–703. The HS1.09_286–703_ construct was chemically synthesized by Twist Biosciences and cloned into a pET-29b(+) vector with a polyhistidine purification tag at the C-terminus. The plasmid was transformed through electroporation into *E. coli* ArcticExpress (DE3) electrocompetent cells and selected on LB agar plates supplemented with 50 μg/mL kanamycin and 20 μg/mL gentamicin grown at 37 °C. A single colony was used to inoculate 5 mL cultures of LB medium supplemented with 50 μg/mL kanamycin and 20 μg/mL gentamicin for growth overnight at 37 °C. The 5 mL cultures were used to inoculate 1.0 L of fresh LB medium supplemented with 50 μg/mL kanamycin and 20 μg/mL gentamicin and grown at 37 °C to an OD_600_ of ∼0.8. The cultures were cooled on ice before induction with 1.0 mM IPTG at 14 °C for 48 h. The cells were harvested by centrifugation at 4 °C. The cell pellet was resuspended in lysis buffer (50 mM triethanolamine pH 8.0, 500 mM NaCl, 1.0 mM DTT, 85 μM n-dodecyl-β-maltoside (DDM), 20 mM imidazole) and 1.0 mg of DNase was added before sonication on ice. The insoluble fraction of the lysate was removed by centrifugation at 4 °C. The supernatant solution was passed through a 0.45 μm filter before loading onto a prepacked 5 mL HisTrap HP nickel affinity column and monitoring the absorbance at 280 nm. The protein was eluted with a 30-column volume gradient of elution buffer (50 mM triethanolamine pH 8.0, 500 mM NaCl, 1.0 mM DTT, 85 μM DDM, 500 mM imidazole). The fractions containing HS1.09_286–703_ were pooled, and excess imidazole was removed by dialysis (50 mM triethanolamine pH 8.0, 500 mM NaCl, 1.0 mM DTT, 85 μM DDM). HS1.09_286–703_ samples were flash frozen in liquid nitrogen and stored at −80 °C. Approximately 2 mg of purified protein was obtained per liter of cell culture.

### Initial Characterization of HS01.09_286–703_

Modified d-galactosides (**3a** and **4**) were tested at a concentration of 5.0 mM as potential acceptor substrates using 25 μM HS1.09_286–703_ with 2.0 mM CDP-glycerol (**2a**) as the donor substrate at 25 °C. Formation of the product CMP was monitored at 280 nm as a function of time after separating the substrates/products by anion exchange chromatography. The reactions were conducted in 50 mM HEPES, pH 8.0, and 3.0 mM MgCl_2_.

### Preparation of Reaction Products **5a** and **5b**

The products of the reaction between CDP-glycerol (**2a**) and methyl-α-d-galactoside (**3a** or **3b**) catalyzed by HS1.09_286–703_ were isolated by anion exchange chromatography. The reaction was initiated by the addition of 20 μM enzyme with 9.0 mM CDP-glycerol (**2a**) and 30 mM of unlabeled (**3a**) or labeled (**3b**) methyl-α-d-galactose in a final reaction volume of 1.0 mL in 50 mM NH_4_HCO_3_, pH 8.0, and 8.0 mM MgCl_2_ for 24 h at 25 °C. The enzyme was removed by filtration, diluted to 15 mL and then applied to a HiTrap Q HP anion exchange column. The product (either **5a** or **5b**) was eluted from the column with a gradient of 500 mM NH_4_HCO_3_, pH 8.0. The fractions containing the desired product were identified by ^31^P NMR spectroscopy.

### Purification of Truncated HS1.09 Containing the Polymerizing Glycerol-phosphate and Galactose Transferase Domains

The gene for HS1.09 (UniProt: Q5M6N7) was purchased from GenScript to include the two putative catalytic functional domains but deleted the first 91 amino acids from the N-terminus. A C-terminal poly histidine purification tag with a TEV cleavage site was added to the HS1.09_92–1095_ construct and subsequently cloned into a pMAL-c5X vector from New England Biolabs (NEB). The pMAL-c5X vector contains an ampicillin resistance gene and fuses an N-terminal maltose binding protein (MBP) tag to HS1.09_92–1095_ with a flexible linker and a factor Xa protease cleavage site. The plasmid containing the gene for HS1.09_92–1095_ was transformed through electroporation into *E. coli* ArcticExpress (DE3) electrocompetent cells and selected on LB agar plates supplemented with 50 μg/mL kanamycin and 20 μg/mL gentamicin grown at 37 °C. A single colony was chosen and inoculated in 5 mL cultures of LB medium supplemented with 50 μg/mL kanamycin and 20 μg/mL gentamicin for growth overnight at 37 °C. The 5 mL cultures were used to inoculate 1.0 L of fresh LB medium supplemented with 50 μg/mL kanamycin and 20 μg/mL gentamicin and grown at 37 °C to an OD_600_ of ∼0.8. The cultures were cooled on ice before induction with 1.0 mM IPTG and subsequent incubation at 14 °C for 48 h. The cell pellet was resuspended in lysis buffer (50 mM triethanolamine pH 8.0, 500 mM NaCl, 1.0 mM DTT, 20 mM imidazole), 1.0 mg of DNase, and the protease inhibitor before sonication on ice. The insoluble fraction of the lysate was removed via centrifugation at 4 °C. The supernatant solution was passed through a 0.45 μm filter before loading onto a prepacked 5 mL nickel affinity column. The protein was eluted with a 30-column volume gradient of elution buffer (50 mM triethanolamine pH 8.0, 500 mM NaCl, 1.0 mM DTT, 500 mM imidazole). The fractions containing HS1.09_92–1095_ were pooled and concentrated to 15 mL in a 20 mL (30 kDa MWCO) concentrator. The sample of HS1.09_92–1095_ was loaded onto a 5 mL MBPTrap HP affinity column equilibrated with 50 mM triethanolamine pH 8.0, 500 mM NaCl. The protein was eluted with a solution containing 50 mM triethanolamine pH 8.0, 500 mM NaCl, 10 mM maltose over a 30-column volume gradient. The fractions containing HS1.09_92–1095_ were pooled, concentrated, flash frozen in liquid nitrogen and stored at −80 °C. Approximately 1.5 mg of purified protein was obtained per liter of cell culture. The amino acid sequence of the isolated protein is presented in Figure S1c and the enzyme will subsequently be denoted as HS1.09. The maltose-binding protein was not removed prior to functional characterization.

### Preparation and Isolation of Product **5b** Using HS1.09

The reaction was catalyzed using 5.0 μM of HS1.09 with 10 mM CDP-glycerol (**2a**) and 8.0 mM of the ^13^C-labeled methyl-α-galactoside (**3b**) in a final reaction volume of 1.0 mL. The reaction contained 50 mM NH_4_HCO_3_, pH 8.0, in a volume of 1.0 mL and was incubated at 25 °C. After 24 h the enzyme was removed by filtration and the solution diluted to 15 mL. The product **5b** was purified using a 5 mL HiTrap Q HP anion exchange column with a gradient of 500 mM NH_4_HCO_3_, pH 8.0. The fractions containing the product **5b** were identified by ^31^P NMR spectroscopy.

### Preparation and Isolation of Reaction Products **6a** and **6b** (Trimer)

The reaction was catalyzed using 5.0 μM of HS1.09 with 9.0 mM CDP-glycerol (**2a**) and 40 mM of 2-glycerol-galactose (**4**) in a final volume of 1.0 mL containing 50 mM NH_4_HCO_3_, pH 8.0 at 25 °C. After 24 h, the reaction was terminated by removal of the enzyme by filtration and subsequently diluted to 15 mL. The product **6a** was purified by anion exchange chromatography using a gradient of 0–500 mM NH_4_HCO_3_. The ^13^C-labeled reaction product **6b** was synthesized using 5.0 μM HS1.09 with 9.0 mM CDP-[2-^13^C]-glycerol (**2b**) and 30 mM 2-glycerol-galactose (**4**) in a final volume of 1.0 mL. The product **6b** was isolated using the same procedure as for product **6a**.

### Preparation and Isolation of Reaction Tetrameric Products **7a-1** and **7b-1**

The reaction was catalyzed using 1.0 μM of HS1.09 with 7.0 mM UDP-Gal and 7.0 mM of the **6a** trimer in a final reaction volume of 1.0 mL containing 50 mM NH_4_HCO_3_, pH 8.0, at 25 °C. The reaction was terminated by removal of the enzyme by filtration. After dilution to 15 mL the tetrameric product **7a-1** was purified by anion exchange chromatography as described previously. The ^13^C-labeled product **7b-1** tetramer was synthesized using 1.0 μM HS1.09 with 3.0 mM UDP-Gal and 2.0 mM of the ^13^C-labeled **6b** trimer in a volume of 0.5 mL. The product was purified using the same procedure as described for **7a-1**.

### Preparation and Isolation of Pentameric Product **8a-1**

The multistep synthesis was initiated by the addition of 6.0 μM HS1.09 with 6.0 mM CDP-glycerol (**2a**) and 25 mM of substrate **4** in 50 mM NH_4_HCO_3_, pH 8.0, in a final volume of 2.0 mL at 25 °C and allowed to continue for 24 h. The reaction was supplemented with 10 units/mL of alkaline phosphatase to hydrolyze the CMP product. After the complete consumption of the CDP-glycerol (**2a**) and formation of the *in situ* product **6a**, UDP-galactose (final concentration of 5.0 mM) was added to the reaction mixture in a final reaction volume of 2.5 mL. The mixture was incubated for an additional 1 h at 25 °C with the complete consumption of UDP-galactose (monitored by anion exchange chromatography). Then 4.0 mM of additional CDP-glycerol (**2a**) was added to the reaction mixture containing the *in situ* product **7a-1** tetramer with a final volume of 3.0 mL, and was incubated for an additional 18 h at 25 °C. The final **8a-1** pentameric product was purified by anion exchange chromatography as described previously.

### Preparation and Isolation of **8a-2** Heptamer

The reaction was initiated by the addition of 3.0 μM of HS1.09 with 3.5 mM UDP-galactose and 3.0 mM **8a-1** pentamer in 50 mM NH_4_HCO_3_, pH 8.0 10 units/mL of alkaline phosphatase. The 1.0 mL reaction was incubated for 1 h at 25 °C. After the addition of the glycerol-P moiety to the **8a-1** pentamer and the *in situ* formation of the **7a-2** hexamer, CDP-glycerol (**2**) was added to a final concentration of 3.0 mM. The 1.1 mL reaction was incubated for an additional 18 h at 25 °C. The **8a-2** heptamer product was purified as previously described.

### Preparation of Longer Polymeric Products

Different ratios of donor and acceptor substrates were combined to analyze the distribution of product polymer lengths. All reactions were catalyzed using 1.0 μM of HS1.09 in a reaction buffer composed of 50 mM triethanolamine, pH 7.25 and supplemented with alkaline phosphatase to hydrolyze the products UDP and CMP. The different ratios tested included the following: (a) 2.0 mM CDP-glycerol (**2a**), 4.0 mM UDP-Gal and 2.0 mM **8a-1** pentamer; (b) 2.0 mM CDP-glycerol (**2a**), 2.0 mM UDP-Gal, and 2.0 mM **8a-1** pentamer; (c) 2.0 mM CDP-glycerol (**2a**), 2.0 mM UDP-Gal, and 1.0 mM **8a-1** pentamer; and (d) 2.0 mM CDP-glycerol (**2a**), 2.0 mM UDP-Gal and 0.2 mM **8a-1** pentamer. The 500 μL reactions were incubated overnight at 25 °C. Samples were heat denatured at 100 °C for 1.0 min and centrifuged to remove the denatured protein. Aliquots were analyzed using ESI-MS to detect the different sized polymers. Table S1 provides a list of predicted masses for polymers up to **7a-8**.

### Preparation of Longer ^13^C-Labeled Products

To help confirm the identity of the product distribution for longer polymers, experiments were conducted using the ^13^C-labeled CDP-glycerol (**2b**). The reactions were initiated using 1.0 μM HS1.09 with 2.0 mM CDP-glycerol (**2b**), 2.0 mM UDP-Gal, and 0.2 mM of the unlabeled pentamer **8a-1** in 50 mM triethanolamine pH 7.25. The 500 μL reaction was incubated overnight at 25 °C and terminated by heat denaturation at 100 °C for 1.0 min.

### Kinetic Measurements for Addition of Substrates to the Growing Polymeric Chain by HS1.09

The transfer of either galactose from UDP-Gal or glycerol-P from CDP-glycerol to acceptor substrates of varying length catalyzed by HS1.09 was determined by measuring the rate of formation of either CMP or UDP as a function of time. The reactions were conducted in 50 mM triethanolamine buffer at 25 °C in an initial volume of 0.50 mL. At various times 50 μL aliquots were withdrawn, and the reaction quenched by heating the sample to 100 °C in a water bath for 1.0 min and then centrifuged to remove the precipitated enzyme. The amount of CMP or UDP formed as a function of time was quantitated using a HiTrap Q HP anion exchange column to separate the products from the substrates. The substrates and products were separated using a 0–500 mM gradient of KCl in 50 mM MES, pH 6.0. Formation of CMP was monitored at 280 nm, whereas the formation of UDP was monitored at 255 nm. Control experiments were conducted in parallel in the absence of the acceptor substrate to monitor the rate of unproductive hydrolysis of the donor substrate. The enzyme was varied from 100 nM to 25 μM and the concentrations of the two donor substrates, CDP-glycerol and UDP-Gal, were kept constant at 2.0 mM. The concentrations of the five acceptor substrates (**3a**, **4**, **6a**, **7a**, and **8a**) were used at 2.0 mM, except for **4**, which was used at a concentration of 4.0 mM.

## Results and Discussion

### Characterization of HS1.11 as a l-Glycerol-3-P Cytidylyltransferase

A simple NCBI BLAST analysis of HS1.11 (UniProt id: Q5M6N5) demonstrates that this protein is homologous to those enzymes previously annotated as TagD and likely catalyzes the formation of CDP-(2*R*)-glycerol from MgCTP and l-glycerol-3-P. The primary amino acid sequence is 45% identical to that of l-glycerol-3-P cytidylyltransferase (GCT) from *Bacillus subtilis* (UniProt id: P27623) whose three-dimensional crystal structure has previously been determined (PDB id: 1N1D).^26^ The GCT from *C. jejuni* was purified to homogeneity and subsequently shown to catalyze the formation of CDP-(2*R*)-glycerol (**2a**) in the presence of CTP and l-glycerol-3-P. The product was purified by anion exchange chromatography and formation of CDP-(2*R*)-glycerol (**2a**) confirmed by ESI mass spectrometry with an *m*/*z* for the [M – H]^−^ anion of 476.05 (Figure S2a) and ^31^P NMR spectroscopy (Figure S3a). The GCT was also used to prepare CDP-glycerol with a ^13^C-label at C2 of the glycerol moiety (compound **2b**). The product was confirmed by ESI-MS with an *m*/*z* of 477.05 for the [M – H]^−^ anion (Figure S2b) and ^31^P NMR spectroscopy (Figure S3b).

The rate of CDP-glycerol formation catalyzed by GCT from *C. jejuni* was initially tested at fixed concentrations of CTP (2.0 mM) and l-glycerol-3-P (5.0 mM) at pH 8.0. The rate of product formation was determined by quantitatively separating the products and unreacted substrates from one another by anion exchange chromatography. Under these conditions the initial rate of CDP-glycerol formation was 14 ± 1 s^–1^ at 25 °C. Under identical conditions the rate of product formation using ATP and UTP was 1.5 ± 0.1 s^–1^ and 0.53 ± 0.03 s^–1^, respectively. The rate of product formation with GTP was less than 0.01 s^–1^. The *B. subtilis* homologue has a reported turnover number of 19 s^–1^ using CTP and l-glycerol-P as substrate.^30^

### Bioinformatic Analysis of HS1.09

**Figure 3:**
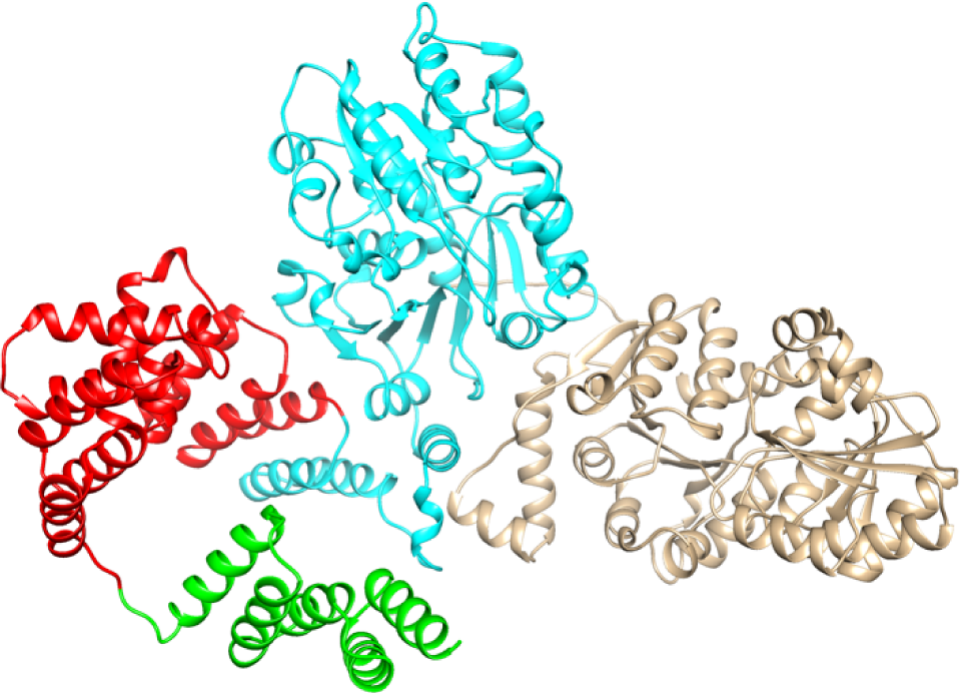
AlphaFold2 prediction of the three-dimensional structure of HS1.09. Residues 1–91 form a multihelical bundle of unknown function (green). Residues 92–285 are denoted as a TPR region (red). Residues 286–703 form a TagF-like glycerol-3-P transferase domain (cyan). Residues 704–1095 fold as a GT4 glycosyltransferase domain (brown).

The AlphaFold2 predicted structure of HS1.09 (UniProt id: Q5M6N7) is presented in Figure 3 and it reveals the formation of multiple folding domains.^25,27,28,31,32^ The first 91 amino acids (colored green in Figure 3) appear to form a multihelix bundle that is observed in many of the proteins found within the enzymes required for CPS formation in *C. jejuni* and *A. pleuropneumoniae*.^25^ The second domain (colored red) extends from residue 92 through 285 and forms what has been described previously as a tetratricopeptide repeat region (TPR).^31^ TPR domains facilitate protein–protein interactions for chaperone activity, oligomerization, or cellular localization, but do not possess a catalytic function.^31^ The third domain (colored blue) extends from residues 286–703 and has a TagF-like structural fold, similar to the enzyme (PDB id: 3L7K) from *Staphylococcus epidermidis* that uses CDP-glycerol to form a repeating polymer of glycerol phosphate (teichoic acid).^27^ The C-terminal domain (residues 704–1095) folds similarly to TarM from *Staphylococcus aureus*, a GT4 glycosyltransferase that transfers *N*-acetyl-d-glucosamine to one of the hydroxyl groups of a polymeric teichoic acid using UDP-GlcNAc.^28^ These comparisons suggest that for the biosynthesis of the repeating copolymer of glycerol-P and d-galactose within the CPS of the HS:1 serotype of *C. jejuni* that the third domain of HS1.09 will likely catalyze the transfer of glycerol phosphate to the d-galactose moiety and that the C-terminal domain will catalyze the transfer of d-galactose to the glycerol phosphate moiety. This proposal is further supported by the observation that the C-terminal domain is a GT4 glycosyltransferase, and these enzymes are known to catalyze the transfer of sugars with retention of configuration as observed in the published structure of the HS:1 serotype CPS.^28,33^ To test this hypothesis, plasmids for the third and fourth domains were cloned and expressed in *E. coli* and subsequently tested for catalytic activity.

### Characterization of HS1.09_286–703_

**Figure 4:**
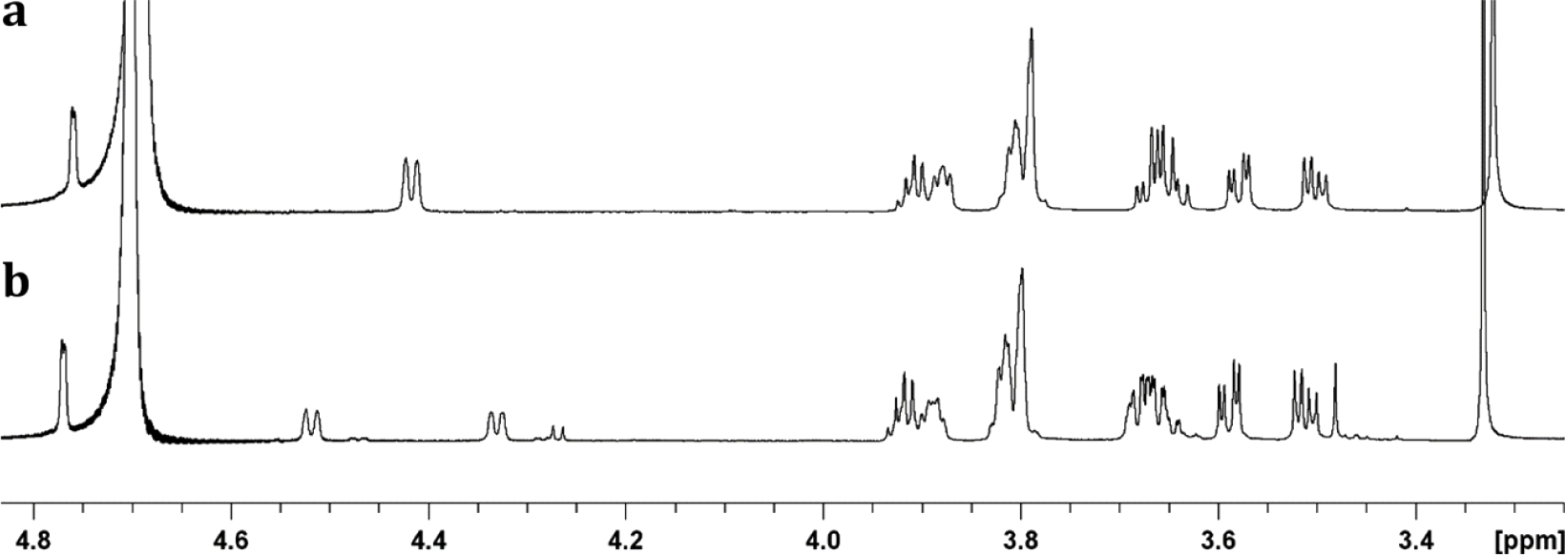
800 MHz ^1^H NMR spectra of products **5a** and **5b**. (a) For product **5a** the doublet (^3^*J*_P–H_ = 9.1 Hz) for the hydrogen at C4 of the galactose moiety appears at 4.425 ppm. (b) For the ^13^C-labeled product **5b** a pair of doublets (^3^*J*_H–P_ = 9.1 Hz and ^2^*J*_H–C_ = 150 Hz) appears at 4.325 and 4.525 ppm for the hydrogen at C4.

The third domain of HS1.09 was purified to homogeneity after expression in *E. coli*. The enzyme was subsequently tested as a catalyst for a reaction between CDP-glycerol (**2a**) and two different modified galactose acceptors. The enzyme was found to be catalytically active with either methyl-α-d-galactoside (**3a**) or 2-glycerol-d-galactoside (**4**) as determined by the formation of CMP via anion exchange chromatography. At a fixed CDP-glycerol concentration of 2.0 mM, and 5.0 mM of either substrate **3a** or **4**, the initial rates of product formation were determined to be 5.9 ± 0.2 and 6.1 ± 0.2 h^–1^, respectively, at pH 8.0 and 25 °C. The product of the reaction between CDP-glycerol (**2a**) and **3a** was isolated by anion exchange chromatography and shown by ESI mass spectrometry to have a [M – H]^−^ anion of 347.08, fully consistent with the formation of compound **5a** (Figure S2c). The ^1^H NMR spectrum of the isolated product is shown in Figure 4a where the resonance for the hydrogen at C4 of **5a** appears as a doublet at 4.425 ppm with a coupling constant (^3^*J*_P–H_) to the adjacent phosphorus of 9.1 Hz.

**Scheme 1:**
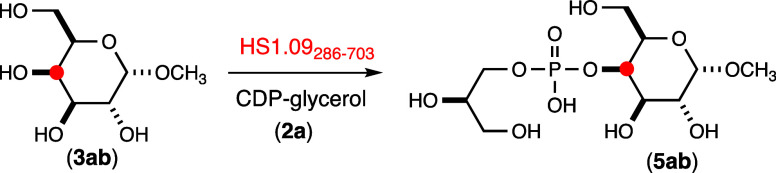
Reaction Catalyzed by HS1.09_286–703_

To confirm that the reaction has occurred at C4 of the galactose moiety, the reaction was repeated with the ^13^C-labeled substrate (**3b**). The product **5b** was isolated by anion exchange chromatography and shown to have a *m*/*z* of 348.08 for the [M–H]^−^ anion, consistent with the ^13^C substitution at C4 (Figure S2d). The ^1^H NMR spectrum of the isolated product (Figure 4b) also exhibits an additional coupling (^1^*J*_H–C_) of 150 Hz between the ^13^C-label at C4 with the hydrogen at C4 of the galactose moiety. The ^31^P NMR spectra for **5a** and **5b** are shown in Figure S4 where the introduction of the ^13^C-label in the product **5b** exhibits an additional phosphorus–carbon coupling constant of 6.1 Hz (^2^*J*_P–C_) with the phosphoryl group, consistent with the addition of the phosphoryl group at C4 of the galactose moiety. The overall reaction is shown in Scheme 1.

### Characterization of HS1.09

Attempts to express and purify the putative galactosyl transferase domain (residues 701–1095 and 715–1095) of HS1.09 failed. We therefore tried to express the gene for the entire protein minus the first 91 amino acids. To facilitate better expression and solubility, we appended the new construct to the maltose binding protein at the N-terminus. This construction was successful, and it enabled us to purify HS1.09 that contained both the glycerol-P and galactosyltransferase domains of the putative glycosyl polymerase. Our first attempt at the functional characterization of the full length HS1.09 was formation of the **6a** trimer from acceptor substrate **4** using CDP-glycerol (**2a**) as the glycerol-P donor. Incubation of HS1.09 in the presence of **2a** and **4** for 24 h resulted in the complete consumption of the CDP-glycerol (**2a**) and formation of product **6a**. The trimeric **6a** product was isolated by anion exchange chromatography and structurally characterized by ESI mass spectrometry, and ^1^H and ^31^P NMR spectroscopy. An *m*/*z* of 407.09 was obtained by ESI-MS, consistent with the expected elemental composition for **6a** and an *m*/*z* of 408.09 was obtained for the corresponding ^13^C-labeled product (**6b**) when **2b** was substituted for **2a** (Figure S5). The ^1^H and ^31^P NMR spectra for product **6a** are presented in Figure S6.

**Scheme 2:**
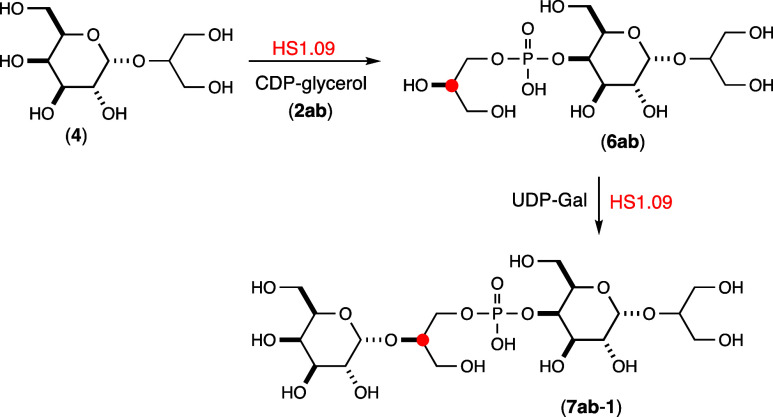
Reactions Catalyzed by HS1.09

**Figure 5:**
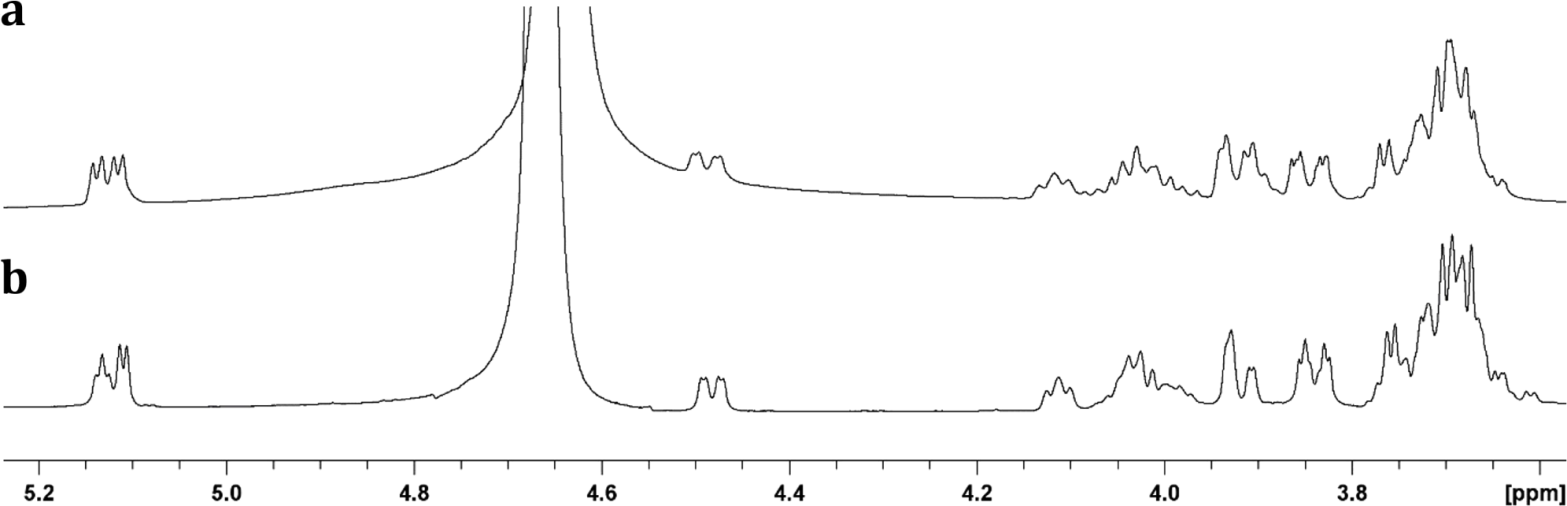
^1^H NMR spectra of the unlabeled tetramer (**7a-1**) and the corresponding ^13^C-labeled product **7b-1**. (a) The two doublets for the anomeric protons of the galactose moieties appear at 5.12 ppm for product **7a-1**. (b) The resonance at 5.14 ppm for the anomeric proton from the second galactose moiety of product **7b-1** is an unresolved triplet due to the additional coupling to the ^13^C-label at C2 of the glycerol moiety.

The isolated **6a** trimeric product was subsequently used to synthesize the anticipated tetrameric product **7a-1** using UDP-Gal as the sugar donor. HS1.09 was incubated with 7.0 mM each of the **6a** trimer and UDP-Gal for 2 h. The product was isolated by anion exchange chromatography and subjected to ESI-MS and NMR spectroscopic analyses. The *m*/*z* for the isolated product was 569.15, fully consistent with the expected mass for tetramer **7a-1** (Figure S5c). The ^31^P NMR spectrum (Figure S7a) shows a single resonance at ∼1.05 ppm and the ^1^H NMR spectrum (Figure 5a) shows an additional resonance at ∼5.12 ppm for the C1 hydrogen of the newly added galactose moiety. The ^13^C-labeled tetramer **7b-1** was synthesized in the same manner except that the ^13^C-labeled CDP-glycerol (**2b**) was used instead of **2a**. ESI-MS analysis of the isolated product **7b-1** provided an *m*/*z* of 570.15, consistent with the addition of a single ^13^C-label to the tetramer (Figure S5d). The ^1^H NMR spectrum of **7b-1** is presented in Figure 5b and it clearly shows the additional coupling of the ^13^C-label at C2 of the glycerol moiety and the anomeric hydrogen from the newly added galactose moiety of the tetramer. The ^31^P and ^13^C spectra for tetramer **7b-1** exhibit doublets with couplings constants of 7.5 Hz for ^3^*J*_C–P_ (Figure S7b,c). These experiments clearly demonstrate that the full length HS1.09 catalyzes the transfer of both glycerol-P and galactose to the growing polymeric chain and that the linkage between the galactose and glycerol moieties is via C2 of the glycerol moiety. These reactions are summarized in Scheme 2.

### Synthesis of Pentameric (**8a-1**) and Heptameric (**8a-2**) Oligomers

The pentameric product (**8a-1**) was synthesized for further product characterization and as the initiating primer for the preparation of longer polysaccharide products. The reaction was initiated by the addition of HS1.09, CDP-glycerol, and compound **4** until the consumption of the CDP-glycerol was complete. One equivalent of UDP-Gal was added to the *in situ* prepared trimer **6a** and allowed to incubate for 2 h to form the tetramer **7a-1** with the complete consumption of the added UDP-Gal. In the final step another equivalent of CDP-glycerol (**2a**) was added and allowed to incubate for 18 h. The pentameric product (**8a-1**) was purified by anion exchange chromatography and characterized by ^1^H and ^31^P NMR spectroscopy (Figure S8). The ^31^P NMR spectrum exhibited a single resonance for each phosphorus at 1.05 and 1.10 ppm (Figure S8a). The ^1^H NMR spectrum (Figure S8b) showed the appearance of a doublet for each of the anomeric protons from the two galactose moieties contained within the pentameric product (**8a-1**). The composition of the isolated pentamer **8a-1** was confirmed by the identification of an *m*/*z* of 723.15 and 361.07 for the *z* = 1 and *z* = 2 anions, respectively (Figure S9a).

The isolated pentamer (**8a-1**) was used as the starting point for the synthesis of the heptamer (**8a-2**). In this case HS1.09 was incubated with the isolated pentamer (**8a-1**) and UDP-Gal in a 1:1 ratio for 2 h. After complete consumption of the UDP-Gal, an additional equivalent of CDP-glycerol was added, and the reaction allowed to continue for an additional 18 h. The product was isolated by anion exchange chromatography and the reaction product confirmed using ESI-MS to demonstrate the appearance of peaks at an *m*/*z* of 1039.20 and 519.10 for the *z* = 1 and *z* = 2 anions (Figure S9b).

### Synthesis of Longer Polysaccharides

Longer polysaccharides were synthesized by incubation of HS1.09 with variable concentrations of UDP-Gal, CDP-glycerol, and the pentameric primer. In the first instance, each of two donor substrates and the primer were added at a concentration of 2.0 mM. The reaction was initiated by the addition of enzyme and the reaction allowed to continue for 24 h when it was terminated by heat denaturation of the enzyme. ESI-MS analysis of the product mixture clearly showed primary formation of the heptameric product (**8a-2**) with peaks identified for the [M-H]^−^ (*m*/*z* = 1039.20) and [M – 2H]^2–^ (*m*/*z* = 519.10) anions (Figure S10).

In the second instance, the reaction was started using an initial concentration of UDP-Gal (4.0 mM) that was twice the concentration of CDP-glycerol and the primer (2.0 mM each). As expected, most of the oligomers that were detected by ESI-MS were capped with d-galactose. The hexamer (**7a-2**) was identified with peaks for the [M – H]^−^ (*m*/*z* = 885.20) and [M – 2H]^2–^ (*m*/*z* = 442.09) anions while the octamer (**7a-3**) was detected by peaks for the [M – H]^−^ (*m*/*z* = 1201.26) and [M – 2H]^2–^ (*m*/*z* = 600.12) anions. Peaks for the presence of the decamer (**7a-4**) were detected for the [M – 2H]^2–^ (*m*/*z* = 758.15) and [M–3H]^3–^ (*m*/*z* = 505.10) anions (Figure S11).

Longer polymers were detected when the concentration of the starting primer (1.0 mM) was reduced by a factor of 2 relative to that of UDP-Gal and CDP-glycerol (2.0 mM). The most abundant peak detected by ESI-MS was for the heptamer (**8a-2**) at an *m*/*z* = 519.10 for the [M–2H]^2–^ anion. Evidence for the [M – 2H]^2–^ anions of the nonomer (**8a-3**), and undecamer (**8a-4**) was obtained by observation of peaks at 677.12, and 835.15, respectively (Figure S12). Even longer polymers were formed when the primer was reduced further to 0.2 mM. In this case ESI-MS demonstrated the formation of the heptamer (**8a-2**), nonamer (**8a-3**), undecamer (**8a-4**), tridecamer (**8a-5**), and pentadecamer (**8a-6**) via the detection of the [*M*-2H]^2–^ anions at *m*/*z* values of 519.10, 677.12, 835.15, 993.18, and 1151.21, respectively (Figure S13). The heptadecamer (**8a-7**) was the longest polymer detected with a prominent [M – 3H]^3–^ anion at an *m*/*z* of 872.49.

To confirm the formation of longer polymers, the ratio containing 0.2 mM primer was repeated using the ^13^C-labeled CDP-glycerol (**2b**) donor. Each new transfer of glycerol-phosphate will add an additional mass unit to the HS1.09 catalyzed polymers causing a predicted shift in the mass spectrum relative to the control experiment using the unlabeled CDP-glycerol (**2a**). The distribution of longer polymers was identical, and the ESI-MS data showed shifted peaks for the heptamer (**8a-2**), nonamer (**8a-3**), undecamer (**8a-4**), tridecamer (**8a-5**), pentadecamer (**8a-6**), and heptadecamer (**8a-7**). The [M – 2H]^2–^ anion peaks were the most abundant for the heptamer (**8a-2**) and nonamer (**8a-3**) at *m*/*z* values of 519.60 and 678.13, respectively. The [M – 3H]^3–^ anion peaks were most prominent for the undecamer (**8a-4**), tridecamer (**8a-5**), pentadecamer (**8a-6**), and heptadecamer (**8a-7**) with *m*/*z* values of 557.44, 663.13, 768.81, and 874.50, respectively (Figure S14).

### Rates for Galactose and Glycerol-P Transfer to Various Acceptors

The rates of galactose and glycerol-P transfer to various acceptor substrates were determined by monitoring the change in concentration of the products, UDP and CMP, via anion exchange chromatography, as a function of time. Under these conditions with relatively short acceptors, the transfer of galactose was significantly faster than the transfer of glycerol-P. In addition, the transfer of either galactose or glycerol-P is faster with a longer acceptor substrate (Table 1). Specifically, the rate for the second galactose transfer was 3.2-fold faster when compared to the first galactose transfer and the l-glycerol-3-P transfer was 32-fold faster with the longer acceptor substrate.

**Table 1:** Rates of Product Formation Catalyzed by HS1.09^a^

Acceptor	Donor	Product	Rate (min^–1^)
dimer (**4**)	CDP-glycerol	trimer (**6a**)	0.23 ± 0.01
trimer (**6a**)	UDP-Gal	tetramer (**7a**)	110 ± 8
tetramer (**7a**)	CDP-glycerol	pentamer (**8a**)	7.4 ± 0.9
pentamer (**8a**)	UDP-Gal	hexamer (**7a-2**)	350 ± 23

a pH 7.25, 25 °C; 2.0 mM of donor and acceptor, except for dimer **4** (4.0 mM).

### Comparison with Csp3D from *A. pleuropneumoniae*

**Figure 6:**
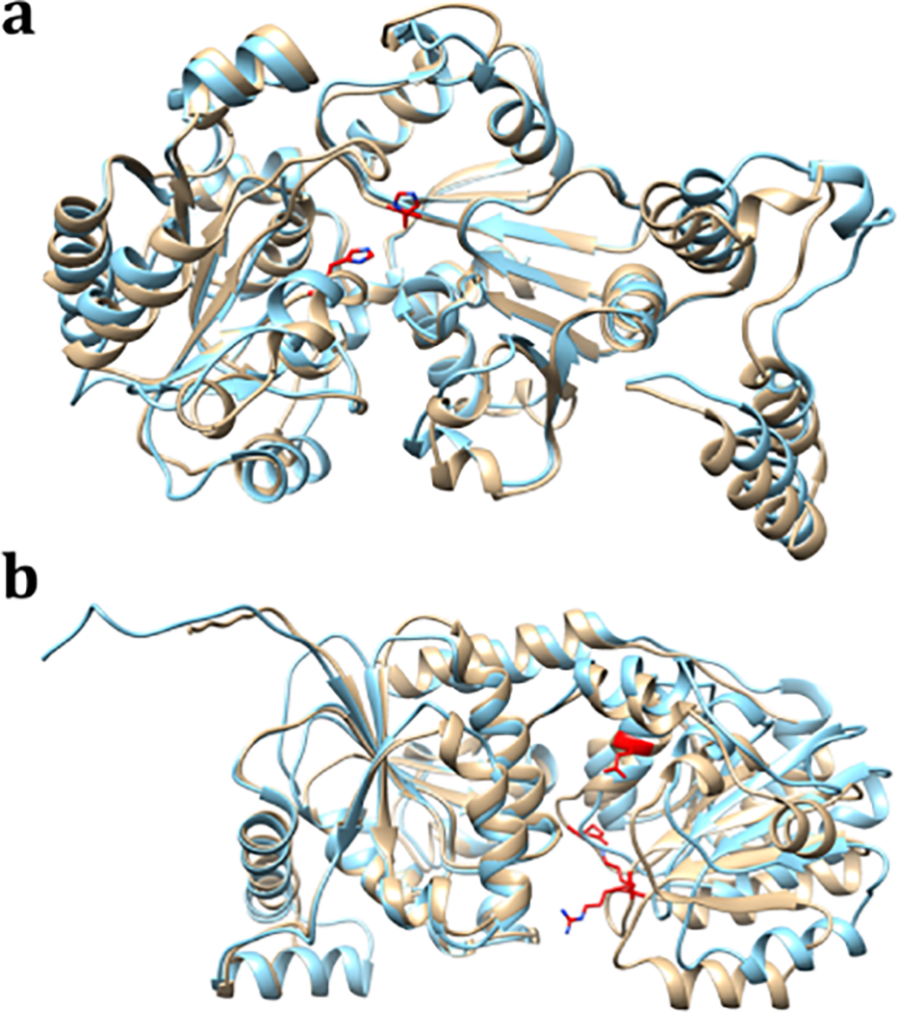
AlphaFold2 generated model of HS1.09 superimposed with Cps3D (PDB id: 8QOY). (a) TagF-like glycerol-3-P transferase domain HS1.09_286–703_ (brown) superimposed with Cps3D_316–735_ (blue). (b) Galactosyl transferase domain of HS1.09_704–1095_ (brown) aligned with Cps3D_736–1138_ (blue). The critical catalytic residues are highlighted in red.

In our investigation of the catalytic properties of HS1.09, we demonstrated that one domain of this enzyme catalyzes the transfer of l-glycerol-3-P from CDP-glycerol to a d-galactose terminated acceptor at C4 and that the second domain catalyzes the transfer of d-galactose from UDP-galactose to the C2 hydroxyl group of a glycerol-P terminated acceptor in alternating fashion to synthesize a repeating polymer of l-glycerol-3P and d-galactose. We were further able to demonstrate that we could initiate polymerization with simple monosaccharides such as the methyl glycoside of d-galactose (**3a**). Recently an enzyme from *A. pleuropneumoniae* (Cps3D) was shown to catalyze the same two reactions as that of HS1.09 and its three-dimensional crystal structure determined to a resolution of 3.0 Å (PDB id: 8QOY).^25^ Overall, the sequence identity between HS1.09 and Cps3D is 37%. Cps3D is a multidomain protein like that proposed for HS1.09 (Figure 3). In Cps3D residues 1–100 are also predicted to form a multihelical bundle that is followed by a TPR domain from residue 100 to 357.^25^ The glycerol-phosphate transferase domain (denoted as CgoT) extends from residue 358–736 and is followed by the galactosyl transferase domain (denoted as CgaT) from residues 748 to 1138.^25^ The CgoT domain of Cps3D is 42% identical to the glycerol-phosphate transferase domain of HS1.09, while the CgaT domain is 43% identical to the galactosyl transferase domain of HS1.09 (data not shown). Cps3D was shown to self-associate as a dimer in solution and computational docking studies were able to identify the probable locations for the binding of CDP-glycerol to the CgoT domain and UDP-gal to the CgaT domain.^25^ A structural overlay of the CgoT domain of Cps3D with that of the AlphaFold2 predicted structure of the glycerol-phosphate transferase domain of HS1.09 identifies H450 and H576 as the two histidine residues shown to be essential for TagF-like enzymes (Figure 6a).^34^ Similarly, the structural overlay of the CgaT domain of Cps3D and the galactosyl transferase domain of HS1.09 identifies R940, K945, E1017 and E1025 as conserved residues within the binding pocket for UDP-Gal (Figure 6b).^25,28^

## Conclusions

The two enzymes required for the polymerization of glycerol-3-phosphate and d-galactose that form the backbone for the capsular polysaccharide of the HS:1 serotype of *C. jejuni* were identified. The central domain of HS1.09 was shown to catalyze the transfer of glycerol-3-phosphate from CDP-glycerol to the C4-hydroxyl group of a d-galactose moiety at the nonreducing end of an acceptor substrate. The C-terminal domain of HS1.09 was shown to catalyze the transfer of d-galactose from UDP-galactose to C2 of glycerol-3-P at the nonreducing end of an acceptor substrate. Acceptor substrates as small as methyl-d-galactoside (**3a**) were efficiently utilized, however longer acceptor substrates were better substrates. ESI mass spectrometry confirmed the identity of oligosaccharides containing at least 17 monomeric units. This investigation will enable the large scale chemoenzymatic synthesis of the repeating polymer of d-galactose and glycerol-phosphate for glycoconjugate vaccine production.

## Supporting Information Available

Amino acid sequences of the protein purified for this investigation, chemical synthesis of various substrates and products, and ESI-MS and NMR spectra of substrates and products (PDF)
