# Supplementary material for: Identification and Characterization of the Two Glycosyltransferases Required for the Polymerization of the HS:1 Serotype Capsular Polysaccharide of Campylobacter jejuni G1
Source: Biochemistry. 2025 Feb 28;64(6):1370–9. doi: 10.1021/acs.biochem.4c00803 (PMC11924219; doi:10.1021/acs.biochem.4c00803)
Supplement: Supplementary file 1 — bi4c00803_si_001.pdf [file bi4c00803_si_001.pdf]

SUPPLEMENTARY INFORMATION

Identification and Characterization of the Two Glycosyltransferases Required for the Polymerization of the HS:1 Serotype Capsular Polysaccharide of *Campylobacter jejuni* G1

Ronnie Bourland[δ], Tamari Narindoshvili[Ψ], and Frank M. Raushel[Ψ,δ,*]

[δ]Department of Biochemistry & Biophysics, Texas A&M University, College Station, TX 77843, United States.

[Ψ]Department of Chemistry, Texas A&M University, College Station, TX 77843, United States.

*To whom correspondence may be addressed: raushel@tamu.edu

(a) HS1.11 with C-terminal polyhistidine purification tag (shown in yellow).

MKNVITFGTFDLFHYGHLRILERAASLGDKLIVGVSSDSLNFAKKHRYPIYSEQERLNIISSLKCVSCVFLEEALEL
KRDYLLKYQANILVMGDDWKGKFDCFNDICDVIYFERTPSISTTEIIERIKLLEHHHHHH

(b) HS1.09$_{286-703}$ with C-terminal polyhistidine purification tag (shown in yellow).

MTHRPFLYFRLCKCLNALEEYKKLSEILSQSQIIQNQPYGLSEDILKDKNLRRRVFYTECYKNLKIIDNMILYESFH
GKSMSCNPYAIFLYLLEQNAFKDFTHIWVVNDLSIVKNKFKKMKNVICVKRGSDLYLKYLASAKYLINNVTFPEYFI
RKEEQKYLNTWHGIPIKYLGKKIKSGFMEHANTQRNFLHATHLIHPNLYTKDILENDYEIKDLFQGQSVLTGYPRVD
LSLKQNAKLKQKLGIKESQKVLLYAPTWRGGLNTQYFDFERLKRDILELKKSNFKVLLSVHHEIKHLFESKLFKDVL
IPSYIEMNELLSIVDVLITDYSSVMFDFMVLERPIICYVYDYEHYKQERGLYFDVDEITHHICKTIEEVKEVLNLEN
LFVKDDLYLTRLKRKFYSLENGKSCERVVSIFFDLEHHHHHH

(c) HS1.09$_{92-1095}$ with N-terminal maltose binding protein purification tag (shown in gray) and C-terminal polyhistidine purification tag (shown in yellow).

MKIEEGKLVIWINGDKGYNGLAEVGKKFEKDTGIKVTVEHPDKLEEKFPQVAATGDGPDIIFWAHDRFGGYAQSGLL
AEITPDKAFQDKLYPFTWDAVRYNGKLIAYPIAVEALSLIYNKDLLPNPPKTWEEIPALDKELKAKGKSALMFNLQE
PYFTWPLIAADGGYAFKYENGKYDIKDVGVDNAGAKAGLTFLVDLIKNKHMNADTDYSIAEAAFNKGETAMTINGPW
AWSNIDTSKVNYGVTVLPTFKGQPSKPFVGVLSAGINAASPNKELAKEFLENYLLTDEGLEAVNKDKPLGAVALKSY
EEELVKDPRIAATMENAQKGEIMPNIPQMSAFWYAVRTAVINAASGRQTVDEALKDAQTNSSSNNNNNNNNNNLGIE
GRISHMQKLWWKEVEDLKKYMQKKGGNFFIYKDLALALENMRRYQEAAKYYELAIKHSKTKDSHLYYKAGFCYERDG
QTDSKLIKYLYANAIKYDDDLNSKILGIGIFHQSNKCWEEANKAYLDFYKYVKNSCSDVLLYNIAYSFEKLFNYQEA
EKYYKKALELNYQECDFHYRLGIVLEKMAKYEEASIYYENTIKRSNTHRPFLYFRLCKCLNALEEYKKLSEILSQSQ
IIQNQPYGLSEDILKDKNLRRRVFYTECYKNLKIIDNMILYESFHGKSMSCNPYAIFLYLLEQNAFKDFTHIWVVND
LSIVKNKFKKMKNVICVKRGSDLYLKYLASAKYLINNVTFPEYFIRKEEQKYLNTWHGIPIKYLGKKIKSGFMEHAN
TQRNFLHATHLIHPNLYTKDILENDYEIKDLFQGQSVLTGYPRVDLSLKQNAKLKQKLGIKESQKVLLYAPTWRGGL
NTQYFDFERLKRDILELKKSNFKVLLSVHHEIKHLFESKLFKDVLIPSYIEMNELLSIVDVLITDYSSVMFDFMVLE
RPIICYVYDYEHYKQERGLYFDVDEITHHICKTIEEVKEVLNLENLFVKDDLYLTRLKRKFYSLENGKSCERVVSIF
FDNVEIRKNIEVCNNILFYTGPFIPNGITNSFKNLIHHLQNSHFNIFVSIDPNSIYSHKERLEQFQLVSENIKVLPR
IGSLNLTLEEFCIEKENLDEEKSLQNYKREFRRLYADVKFKTVINFEGYNVFWVKLFSSVNNNLIFLHNNMQGEFEK
RFPYLEQNFKCYKNYKKILSVSKQTNEQNKKNLAYKYNIAETTFDFLENMINNEDIIEKSKEKLDKKLEKKYFKKDY
KIFINIARLSIEKDQAKLIQAFKVINDKYPKTLLLILGEGPLKEDLEKLIKDLKLDKKVFLLGRIFNPFPYLKKADC
FVMSSNHEGQPMTLLEALVLNKAIVATDIPGNVSVLDNRGGLIVENNVNGLIDGMKKIINRSIEIFYFNTKEYNSQC
LEKLVTFLKSSENLYFQGSSHHHHHH

Figure S1: Protein sequences for the three enzymes purified for this investigation. (a) L-glycerol-3-phosphate cytidylyltransferase (HS1.11). (b) Polymerizing glycerol-P transferase domain of HS1.09$_{286-703}$. (c) Maltose binding protein adduct of HS1.09$_{92-1095}$.

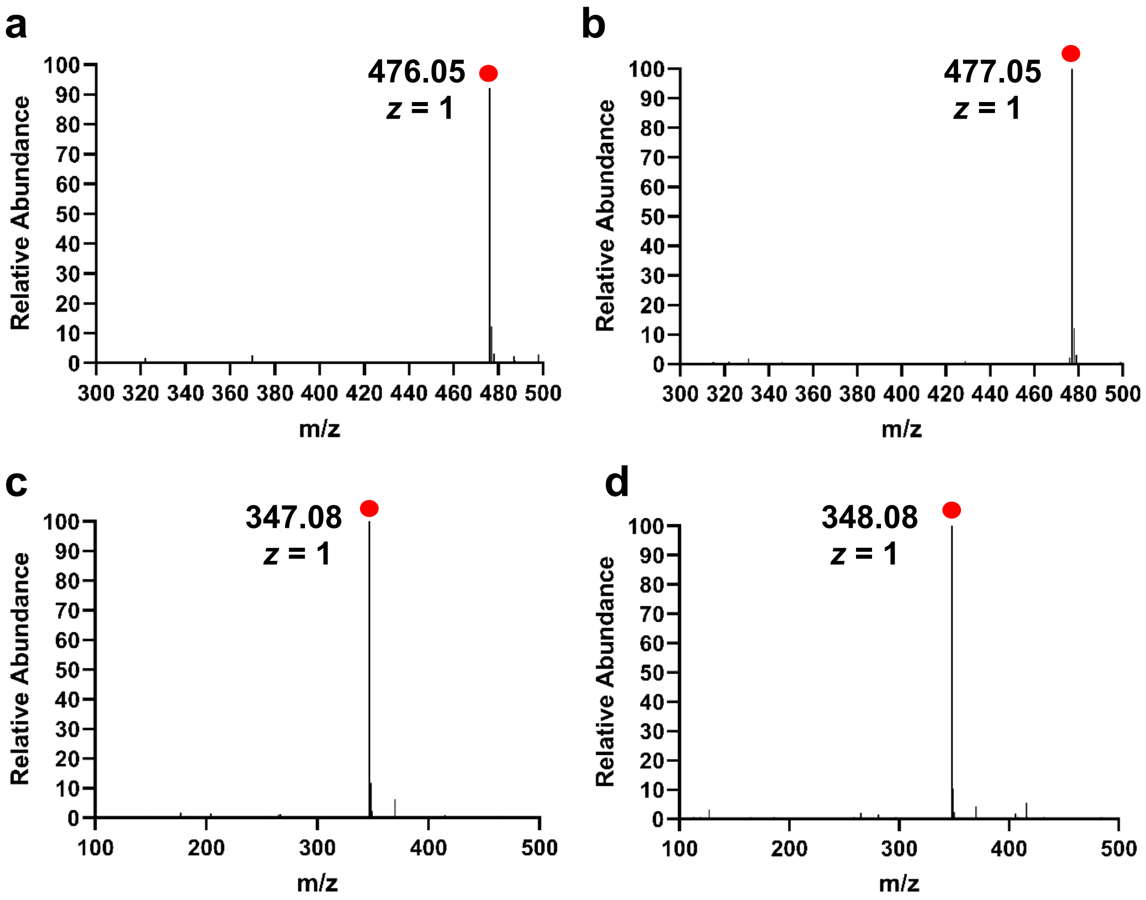

Figure S2. (**a**) ESI-MS (negative mode) of isolated CDP-(2*R*)-glycerol (**2a**) (m/z = 476.05 for z = 1); (**b**) CDP-(2*R*)-[^{13}C-2]-glycerol (**2b**) (m/z = 477.05 for z = 1); (**c**) methyl-α-D-galactose-glycerol-3-phosphate (**5a**) (m/z = 347.08 for z = 1); (**d**) and methyl-α-D-[^{13}C-4]-galactose-glycerol-3-phosphate (**5b**) (m/z = 348.08 for z = 1).

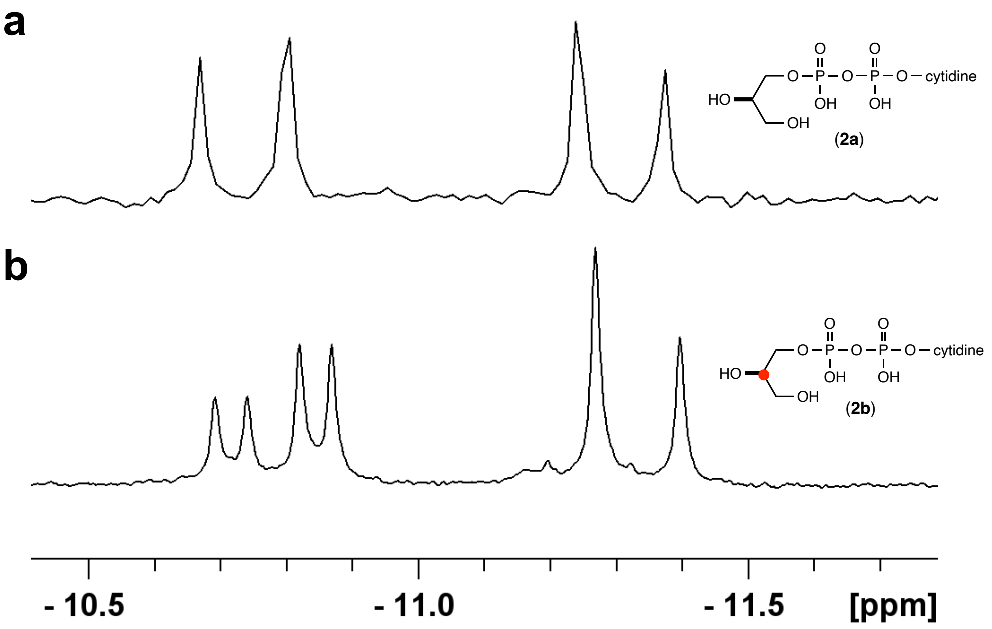

Figure S3. (**a**) ^{31}P NMR spectrum of CDP-(2*R*)-glycerol (**2a**). The doublet for the α-P resonates at -11.3 ppm while the doublet for the β-P resonates at -10.7 ppm with a coupling constant of 21 Hz ($^2J_{P-P}$); (**b**) ^{31}P NMR spectrum of CDP-(2*R*)-[^{13}C-2]-glycerol (**2b**). The double for the α-P resonates at -11.3 ppm while the doublet of doublets for the β-P resonates at -10.8 ppm with coupling constants of 21 Hz ($^2J_{P-P}$) and 8 Hz ($^3J_{C-P}$).

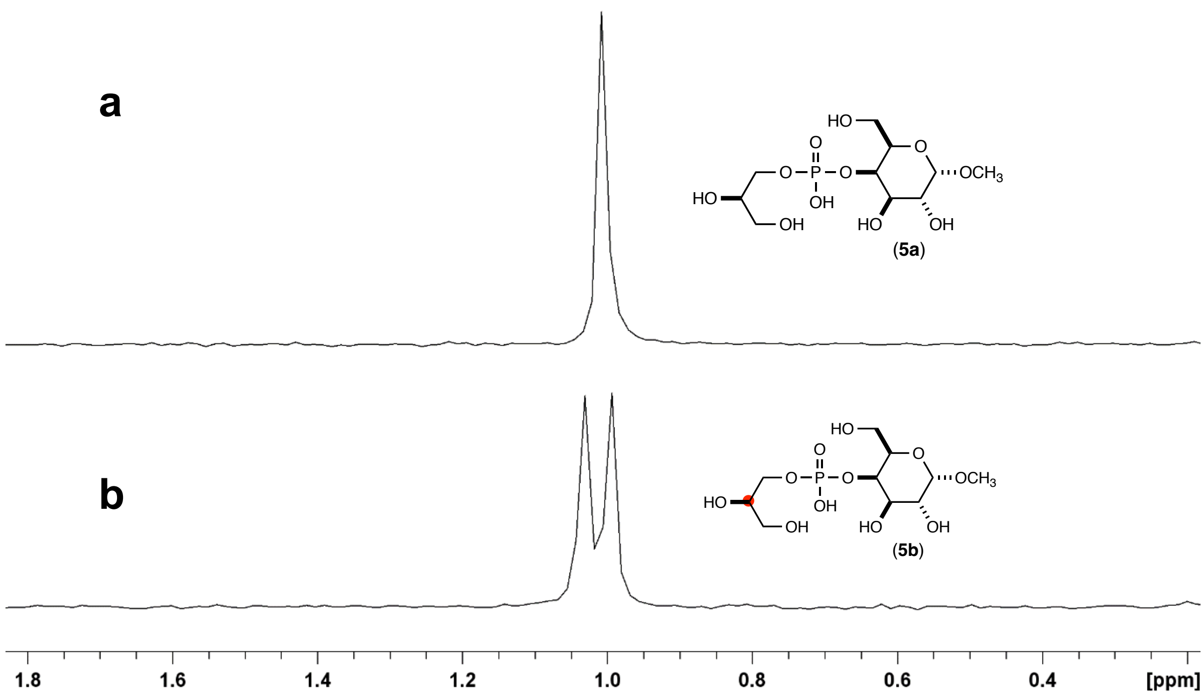

Figure S4. (**a**) Proton-decoupled ^{31}P NMR spectrum of methyl-α-D-galactose-glycerol-3-phosphate (**5a**); (**b**) methyl-α-D-[^{13}C-4]-galactose-glycerol-3-phosphate (**5b**).

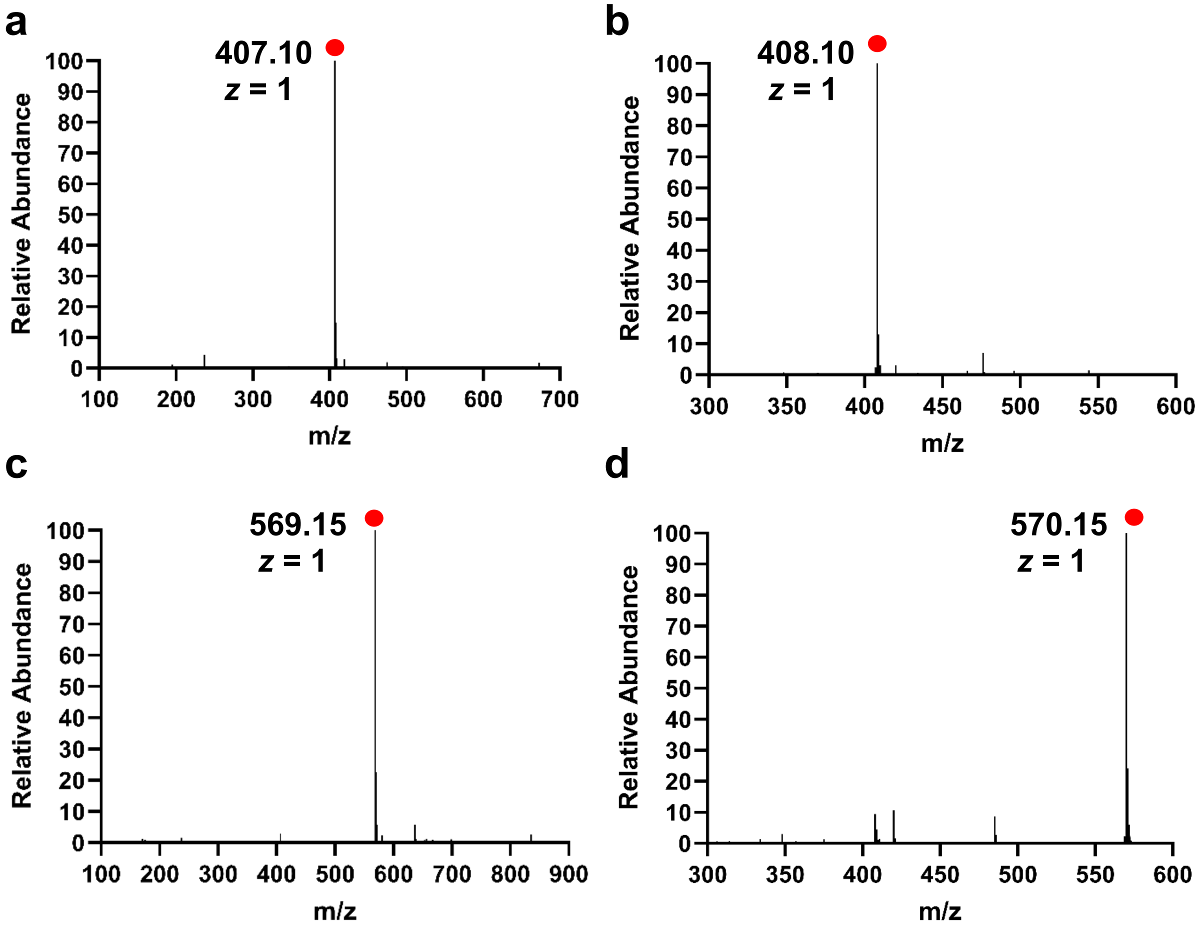

Figure S5. (**a**) ESI-MS (negative mode) of isolated trimer (**6a**) (*m/z* = 407.10 for *z* = 1); (**b**) ^{13}C-labeled trimer (**6b**) (*m/z* = 408.10 for *z* = 1); (**c**) tetramer (**7a-1**) (*m/z* = 569.15 for *z* = 1); and (**d**) ^{13}C-labeled tetramer (**7b-1**) (*m/z* = 570.15 for *z* = 1).

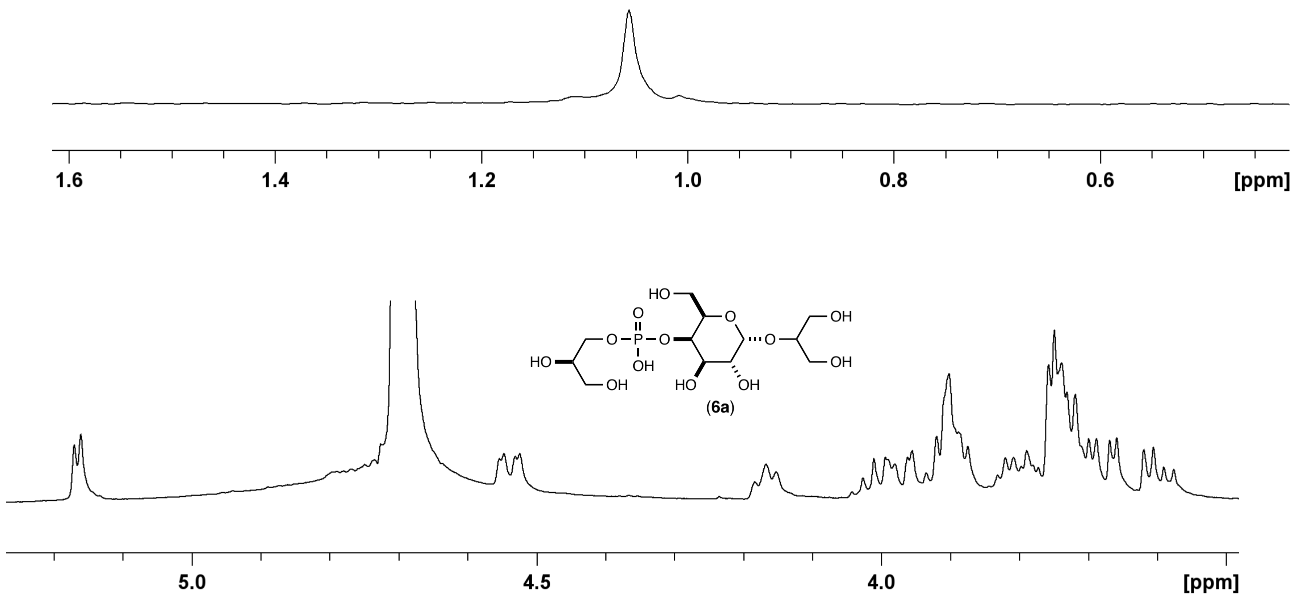

Figure S6: (top) Proton-decoupled ^{31}P-NMR spectrum of compound **6a**. (bottom) ^{1}H-NMR spectrum of isolated trimer (**6a**). The doublet at 5.15 ppm is from the hydrogen at C1 of the galactose moiety while the doublet of doublets at 4.55 ppm is from the hydrogen at C4 of the galactose moiety.

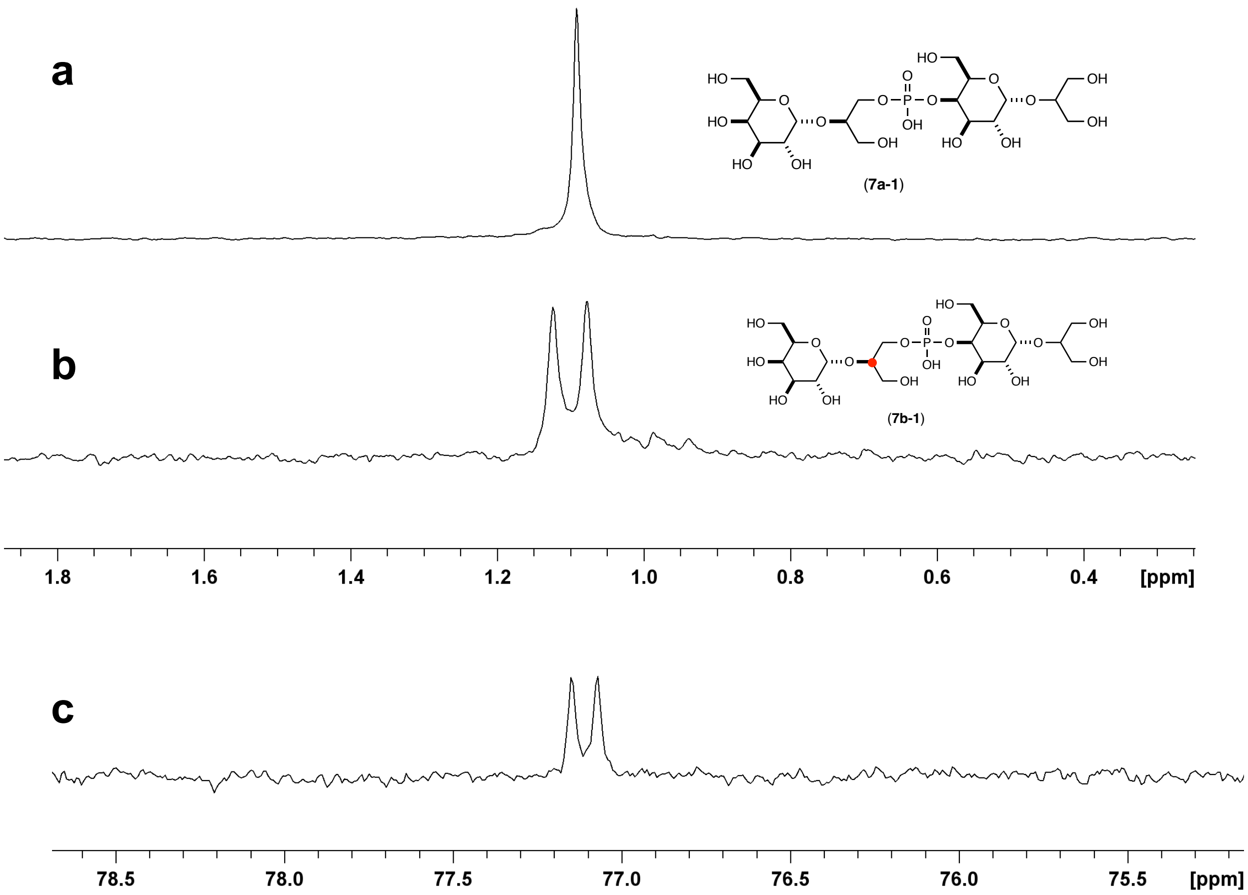

Figure S7. (**a**) ^{31}P NMR spectrum of isolated tetramer (**7a-1**); (**b**) of ^{13}C-labeled tetramer (**7b-1**); (c) ^{13}C NMR spectrum of isolated ^{13}C-labeled tetramer (**7b-1**).

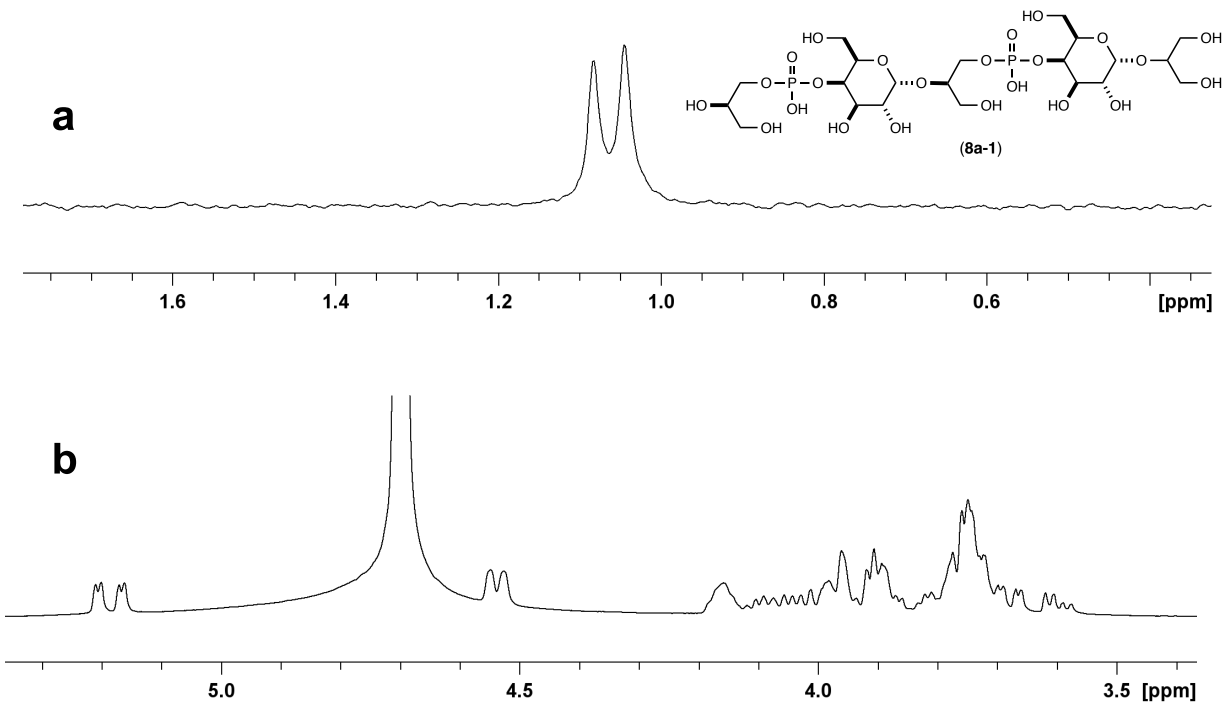

Figure S8. (**a**) ^{31}P NMR spectrum of isolated pentamer (**8a-1**); (**b**) ^{1}H NMR spectrum of isolated pentamer (**8a-1**).

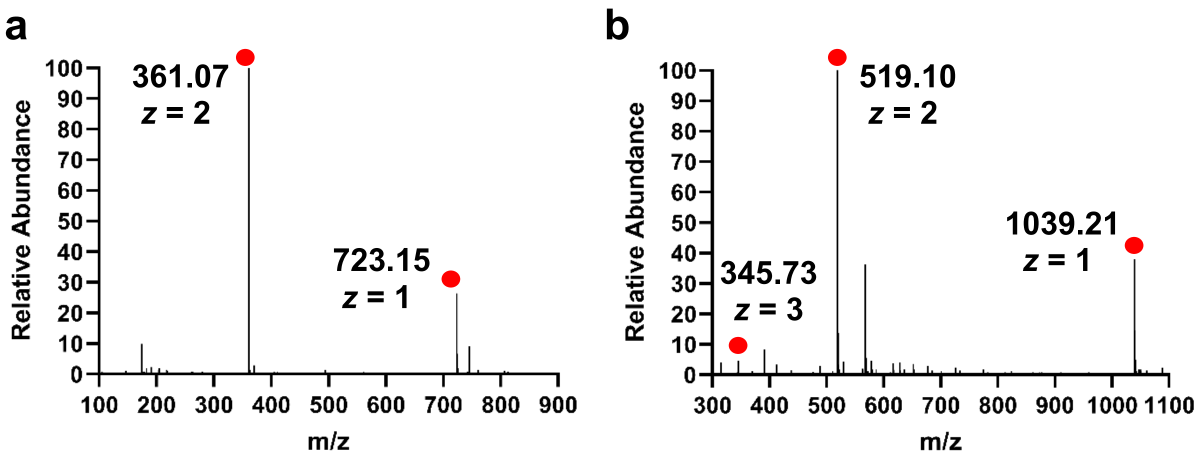

Figure S9. (**a**) ESI-MS (negative mode) for isolated pentamer (**8a-1**) (m/z = 723.15 for z = 1; m/z of 361.07 for z = 2); (**b**) heptamer (**8a-2**) (m/z of 1039.21 for z = 1; m/z of 519.10 for z = 2; and m/z of 345.73 for z = 3).

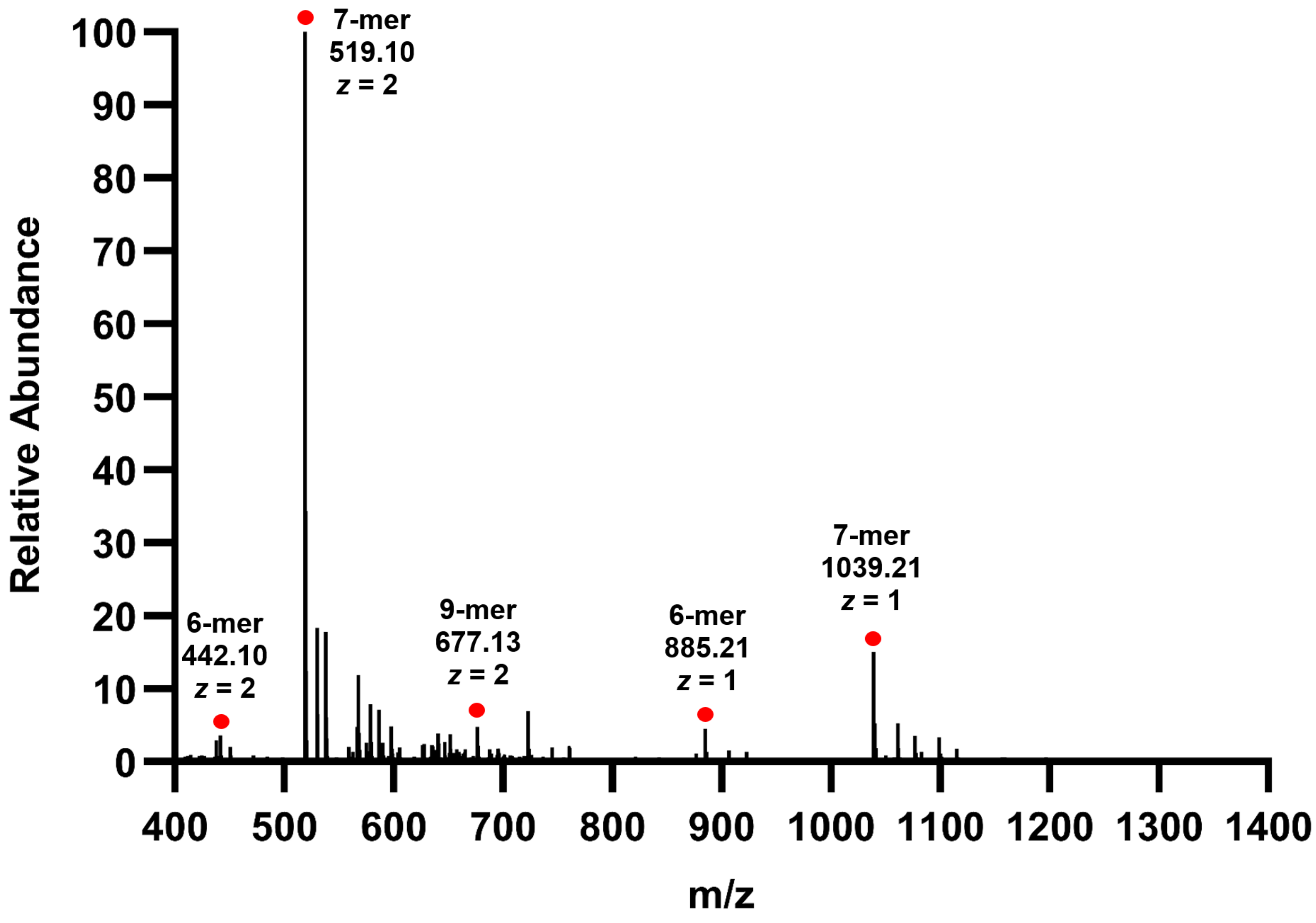

Figure S10. ESI-MS (negative mode) for the products formed using 2.0 mM each of CDP-glycerol, UDP-Gal and primer **8a-1**. Hexamer (**7a-2**) (*m/z* = 885.21 for *z* = 1; *m/z* = 442.10 for *z* = 2), heptamer (**8a-2**) (*m/z* = 1039.21 for *z* = 1; *m/z* = 519.10 for *z* = 2), and nonamer (**8a-3**) (*m/z* = 677.13 for *z* = 2).

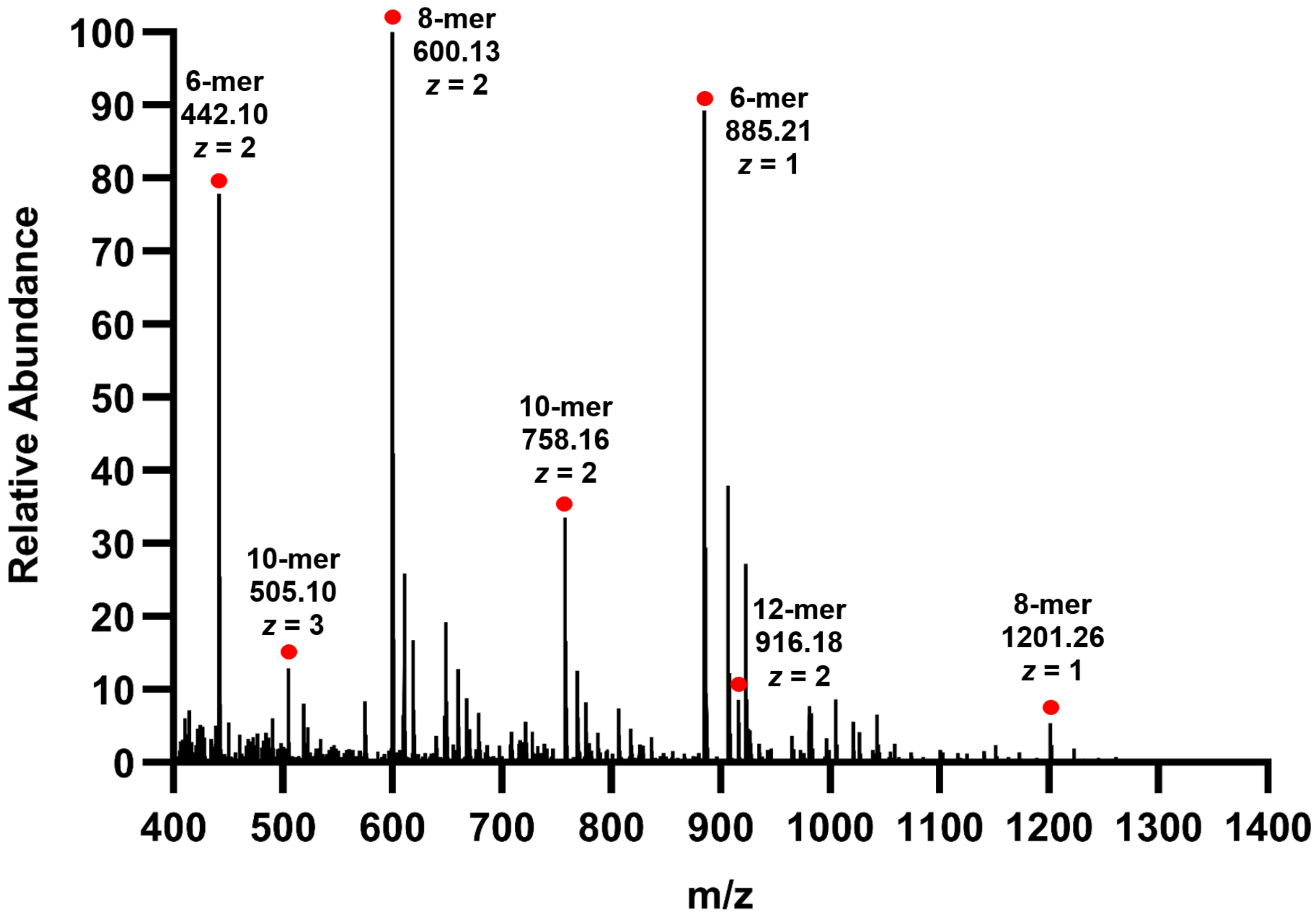

Figure S11. ESI-MS (negative mode) for products formed from the incubation of 2.0 mM CDP-glycerol, 4.0 mM UDP-Gal, and 2.0 mM **8a-1** primer. Hexamer (**7a-2**) (m/z = 885.21 for z = 1; m/z = 442.10 for z = 2), octamer (**7a-3**) (m/z = 1201.26 for z = 1; m/z = 600.13 for z = 2), decamer (**7a-4**) (m/z = 758.16 for z = 2; m/z = 505.10 for z = 3), and dodecamer (**7a-5**) (m/z = 916.18 for z = 2).

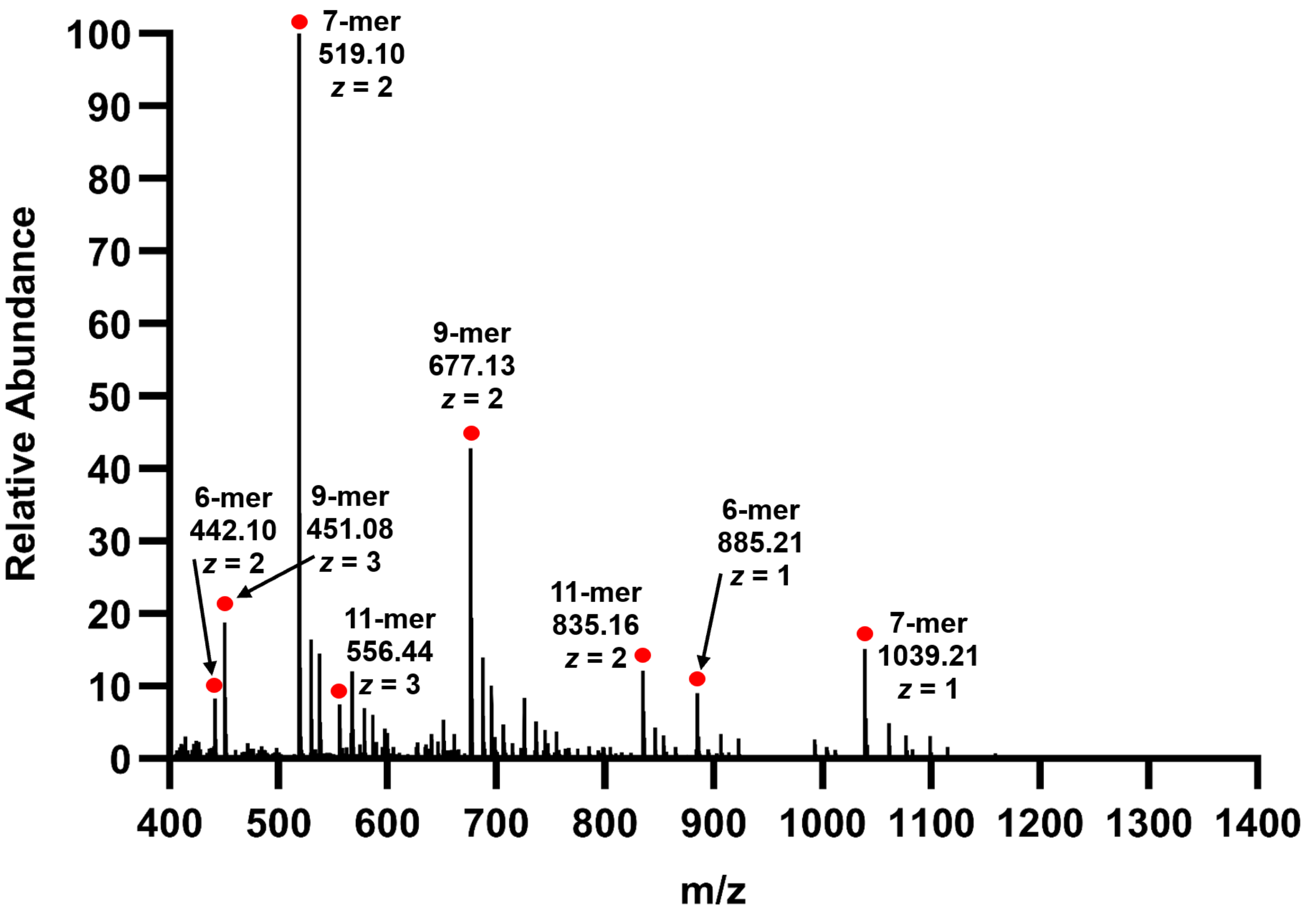

Figure S12. ESI-MS (negative mode) for products formed from the incubation of 2.0 mM each of UDP-Gal and CDP-glycerol with 1.0 mM **8a-1** primer. Hexamer (**7a-2**) (*m/z* = 885.21 for *z* = 1; *m/z* = 442.10 for *z* = 2), heptamer (**8a-2**) (*m/z* = 1039.21 for *z* = 1; *m/z* = 519.10 for *z* = 2), nonamer (**8a-3**) (*m/z* = 677.13 for *z* = 2; *m/z* = 451.08 for *z* = 3), and undecamer (**8a-4**) (*m/z* = 835.16 for *z* = 2; *m/z* = 556.44 for *z* = 3).

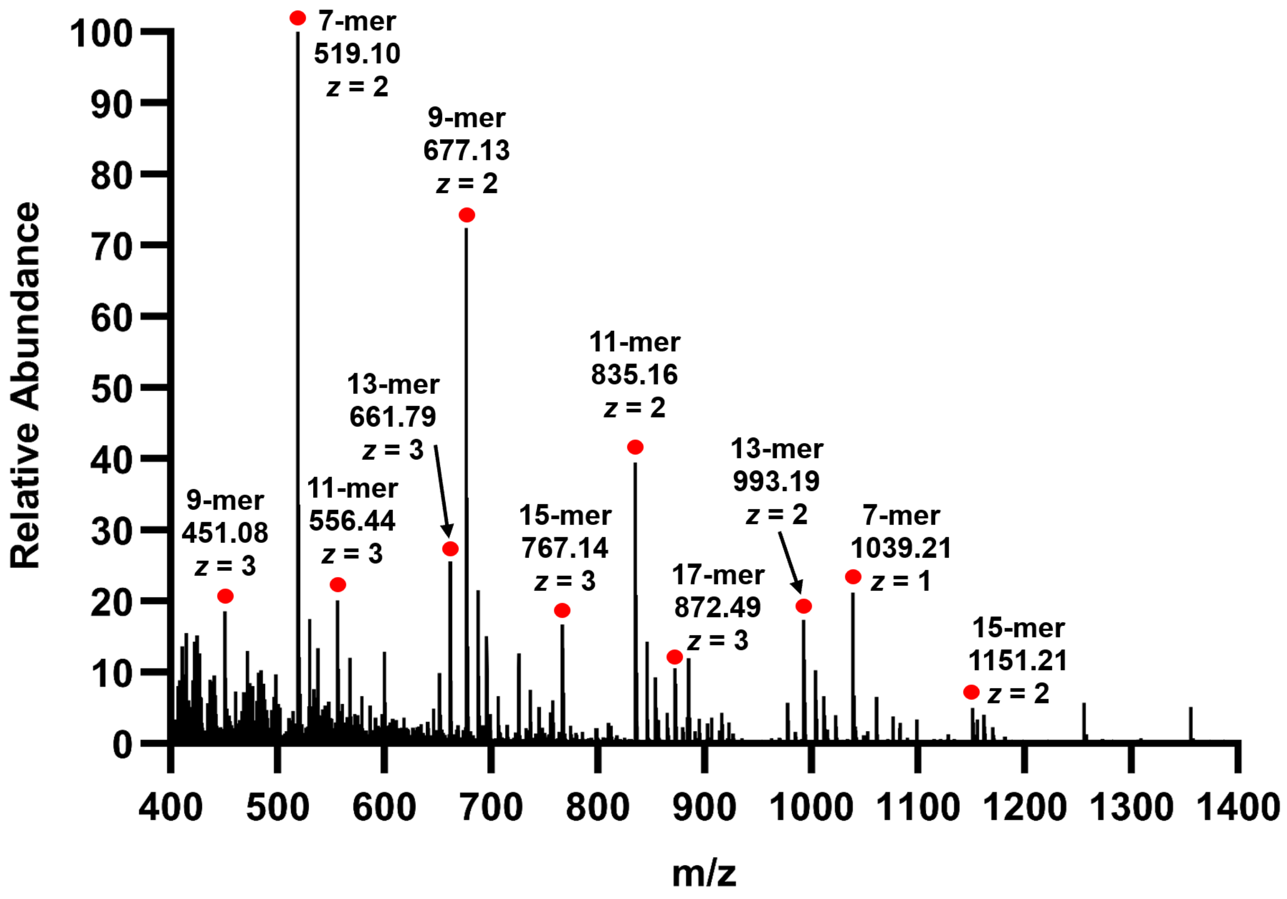

Figure S13. ESI-MS (negative mode) for products formed from the incubation of 2.0 mM each of UDP-Gal, and CDP-glycerol with 0.2 mM **8a-1** promer. Heptamer (**8a-2**) (*m/z* = 1039.21 for *z* = 1; *m/z* = 519.10 for *z* = 2), nonamer (**8a-3**) (*m/z* = 677.13 for *z* = 2; *m/z* = 451.08 for *z* = 3), undecamer (**8a-4**) (*m/z* = 835.16 for *z* = 2; *m/z* = 556.44 for *z* = 3), tridecamer (**8a-5**) (*m/z* = 993.19 for *z* = 2; *m/z* = 661.79 for *z* = 3), pentadecamer (**8a-6**) (*m/z* = 1151.21 for *z* = 2; *m/z* = 767.14 for *z* = 3), and heptadecamer (**8a-7**) (*m/z* = 872.49 for *z* = 3).

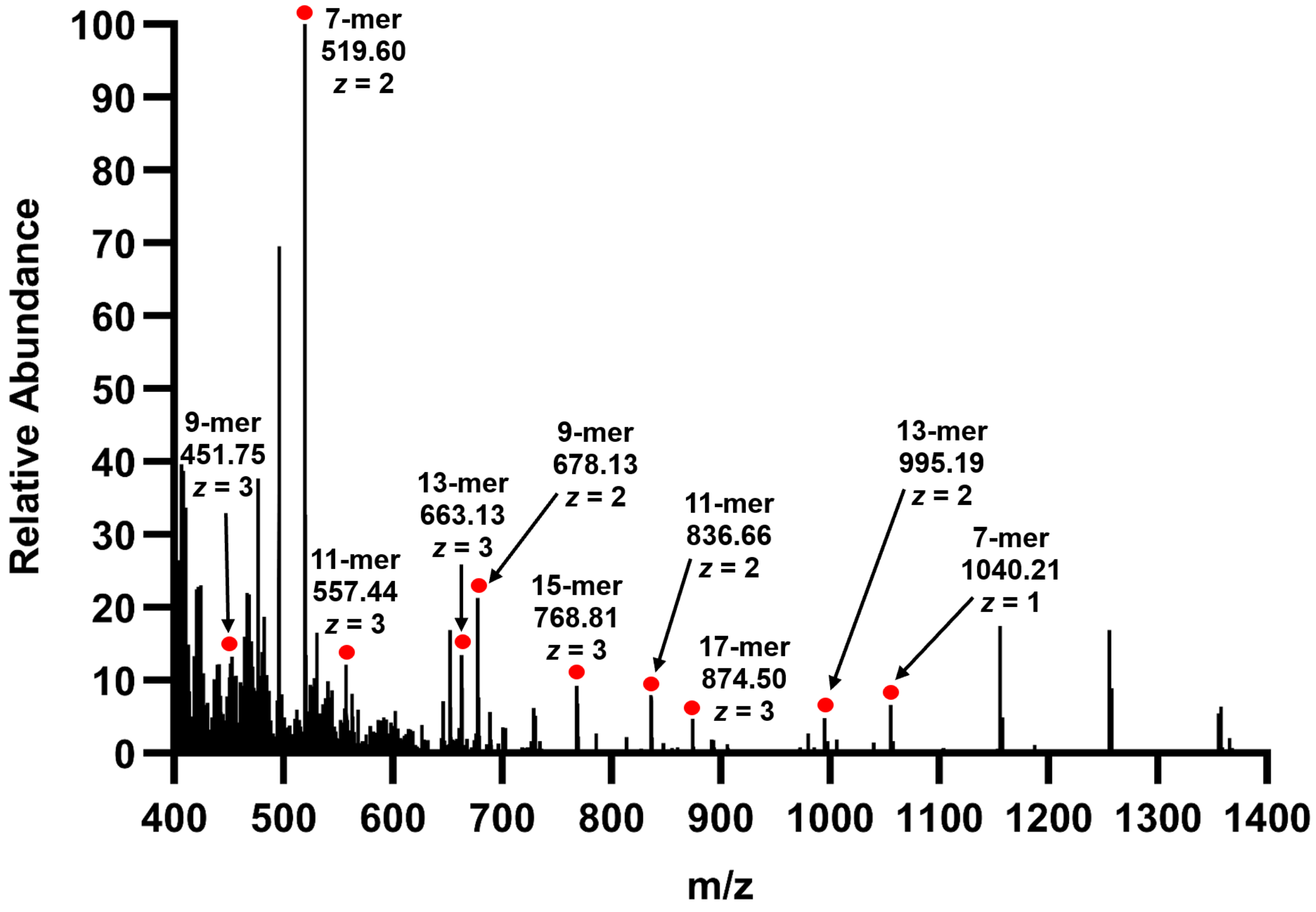

Figure S14. ESI-MS (negative mode) for products formed from the incubation of 2.0 mM UDP-Gal, 2.0 mM CDP-(^{13}C)-glycerol (**2b**) and 0.2 mM **8a-1** primer.

Table S1. Expected mass spectrometry *m/z* values for oligomeric products.

Coded name	**8a-1**	**7a-2**	**8a-2**	**7a-3**	**8a-3**	**7a-4**
Polymer	Pentamer	Hexamer	Heptamer	Octamer	Nonamer	Decamer
z = 1						
$[M-H]^{-1}$	723.15	885.21	1039.21	1201.26	1355.26	1517.32
$[M-2H+Na]^{-1}$	745.15	907.21	1061.21	1223.26	1377.26	1539.32
$[M-2H+K]^{-1}$	761.15	923.21	1077.21	1239.26	1393.26	1555.32
z = 2						
$[M-2H]^{-2}$	361.08	442.11	519.11	600.13	677.13	758.16
$[M-3H+Na]^{-2}$			530.11	611.13	688.13	769.16
$[M-3H+K]^{-2}$			538.11	619.13	696.13	777.16
z = 3						
$[M-3H]^{-3}$			345.74	399.75	451.09	505.11
$[M-4H+Na]^{-3}$					458.42	512.44
$[M-4H+K]^{-3}$					463.76	517.78

Coded name	**8a-4**	**7a-5**	**8a-5**	**7a-6**
Polymer	Undecamer	Dodecamer	Tridecamer	Tetradecamer
z = 1				
$[M-H]^{-1}$	1671.32	1833.37	1987.38	2149.43
$[M-2H+Na]^{-1}$	1693.32	1855.37	2009.38	2171.43
$[M-2H+K]^{-1}$	1709.32	1871.37	2025.38	2187.43
z = 2				
$[M-2H]^{-2}$	835.16	916.19	993.19	1074.22
$[M-3H+Na]^{-2}$	846.16	927.19	1004.19	1085.22
$[M-3H+K]^{-2}$	854.16	935.19	1012.19	1093.22
z = 3				
$[M-3H]^{-3}$	556.44	610.46	661.79	715.81
$[M-4H+Na]^{-3}$	563.77	617.79	669.12	723.14
$[M-4H+K]^{-3}$	569.11	623.13	674.46	728.48

Coded name	**8a-6**	**7a-7**	**8a-7**	**7a-8**
Polymer	Pentadecamer	Hexadecamer	Heptadecamer	Octadecamer
z = 1				
$[M-H]^{-1}$	2303.43	2465.48	2619.49	2797.57
$[M-2H+Na]^{-1}$	2325.43	2487.48	2641.49	2819.57
$[M-2H+K]^{-1}$	2341.43	2503.48	2657.49	2835.57
z = 2				
$[M-2H]^{-2}$	1151.22	1232.24	1309.25	1398.29
$[M-3H+Na]^{-2}$	1162.22	1243.24	1320.25	1409.29
$[M-3H+K]^{-2}$	1170.22	1251.24	1328.25	1417.29
z=3				
$[M-3H]^{-3}$	767.14	821.16	872.50	931.86
$[M-4H+Na]^{-3}$	774.47	828.49	879.83	939.19
$[M-4H+K]^{-3}$	779.81	833.83	885.17	944.53

Synthesis of 2-glycerol-D-galactopyranose (4). For the synthesis of 2-glycerol-D-galactopyranose **4**, commercially available **10** was activated to the α-enantiomer of the trichloroethanimidate derivative **11,** according to a literature procedure [1]. Glycosylation of **11** with benzyl protected glycerol **12** afforded the benzyl protected 2-glycerol-D-galactopyranoside **13,** as a 1:1 mixture of the α- and β-enantiomers. Catalytic hydrogenation of **13** allowed quantitative formation of product **4** as a 1:1 mixture of α- and β-enantiomers (total yield 39%).

Scheme 1: Synthetic scheme for the preparation of compound **4**.

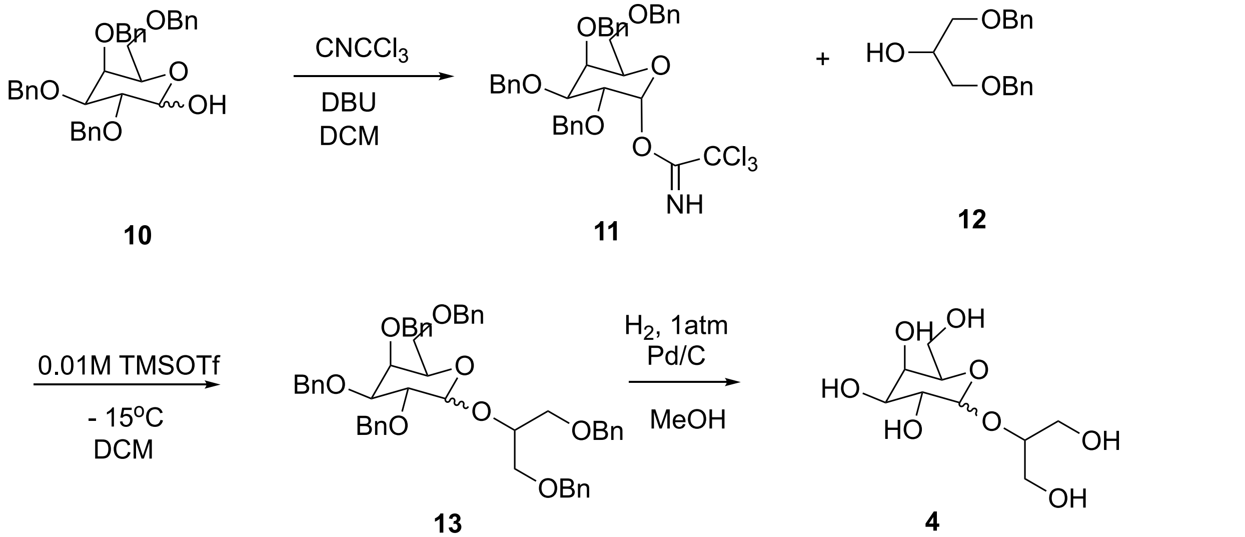

10 11 12 13 4

Step 1: A solution of 2,3,4,6 tetra-O-benzyl galactopyranose **10** (0.71 g, 1.31 mmol, 1.0 equiv.) in anhydrous dichloromethane (15 mL) was cooled to 0 °C and Cl_3CCN (1.5 mL, 15 mmol, 11 equiv.) was added, followed by DBU (0.12 mL). The reaction was stirred at room temperature for 3 h. After concentration, the crude material was subjected to silica gel column chromatography (hexanes:ethylacetate, 10:1) to isolate 0.6 g (yield 67%) of 2,3,4, 6,-tetra-O-benzyl galactopyranose trichloroacetimidate **11** as the α-anomer.

Step 2: 0.6 g (0.88 mmol, 1.0 equiv.) of **11**, 1,3 di-O-benzyl glycerol **12** (0.47 g, 1.73 mmol, 2.0 equiv.) and 4 A° molecular sieves (0.7 g) were suspended in dichloromethane (12 mL) and stirred under argon for 20 min, then the mixture was cooled to -15 °C. Trimethylsilyl trifluoromethanesulfonate (TMSOTf, 0.01 M in DCM, 5 mL) was added dropwise. After 20 minutes, the reaction was quenched with TEA, diluted with dichloromethane, filtered over Celite and concentrated. The crude material was subjected to silica gel column chromatography (hexanes:ethylacetate, 6:1) to isolate 0.51 g (yield 73%) of **13** as a 1:1 mixture of α- and β-anomers.

Step 3: 0.51 g (0.65 mmol) of **13** was dissolved in 40 mL of methanol and 350 mg of Pd/C/10% was added. The mixture was subjected to hydrogen atmosphere and stirred overnight. After filtration, solvent was evaporated to obtain 0.13 g (yield 80%) of **5**, as 1:1 mixture of α- and β-anomers.

2-glycerol-D-galactopyranose (4):

^{1}H NMR (400 MHz, D_2O) δ 5.12 (d, J = 3.8 Hz, 0.5H), 4.50 (d, J = 7.8 Hz, 0.5H), 4.07 (t, J = 6.2 Hz, 0.5 H), 3.98-3.84 (m, 2H), 3.82-3.62 (m, 8H), 3.57-3.49 (m, 0.5 H).

^{13}C NMR (125 MHz, D_2O) δ 80.9, 78.8, 75.2, 72.7, 71.1, 71.0, 69.4, 69.3, 68.7, 68.5, 62.5, 61.6, 61.4, 61.1, 61.0, 60.9, 60.4.

(ESI$^+$) *m/z* $[M + H]^+$ calc. for $C_9H_{19}O_8$: 255.1080, found: 255.1076.

1. Cox, D. J.; Smith, M. D.; Fairbanks, A. J. Glycosylation Catalyzed by a Chiral Bronsted Acid. *Org. Lett.* **2010**, *12*, 1452-1455.

Synthesis of [^{13}C-4]-Methyl-a-D-Galactopyranoside (3b). [^{13}C-4]-D-Galactose (0.095 g, 0.52 mmol) was dissolved in anhydrous methanol (2 mL). Dry Dowex 50WX8 (200-400 mesh) (H$^+$) ion-exchange resin (120 mg) was added and the suspension was refluxed for 24 h. After filtration, the solution was concentrated. Crude product (studied by 13C NMR) was a mixture of methyl α- and β-galactopyranosides and galactofuranosides. To isolate the ^{13}C-labeled methyl-α-D-galactopyranoside, the residue was subjected to silica gel column chromatography (dichloromethane: MeOH, 100:7 to 100:10). The product (25 mg) was isolated as a mixture of α- and β-enantiomers as a 80:20 mixture.

^{1}H NMR (400 MHz, MeOH-d*4*) δ (α- and β-enantiomers) 5.12 (d, J = 3.8 Hz, 0.5H), 4.50 (d, J = 7.8 Hz, 0.5H), 4.07 (t, J = 6.2 Hz, 0.5 H), 3.98-3.84 (m, 2H), 3.82-3.62 (m, 8H), 3.57-3.49 (m, 0.5 H).

^{13}C NMR (125 MHz, MeOH-d*4*) δ (α-enantiomer) 100.1, 70.7, 70.4, 69.7, 69.0, 61.4, 54.2.

(ESI$^+$) m/z $[M + H]^+$ calc. for $C_6{}^{13}CH_{15}O_6$: 196.0902, found: 196.0901.
